# Preventive and Ameliorative Effects of Se- and Zn-Biofortified Chickpeas on MAFLD-Related Metabolic Disturbances

**DOI:** 10.3390/foods15132330

**Published:** 2026-07-01

**Authors:** Emilio López-Millán, Jorge Alberto Uribe-Echeverría, Julián de la Rosa-Millán, Marilena Antunes-Ricardo

**Affiliations:** 1 Tecnologico de Monterrey, School of Engineering and Sciences, Ave. Eugenio Garza Sada 2501 Sur, Col: Tecnologico, Monterrey 64849, NL, Mexico; a01734152@tec.mx (E.L.-M.); a00843448@tec.mx (J.A.U.-E.); juliandlrm@tec.mx (J.d.l.R.-M.); 2 Tecnologico de Monterrey, Institute for Obesity Research, Ave. Eugenio Garza Sada 2501 Sur, Col: Tecnologico, Monterrey 64849, NL, Mexico

**Keywords:** biofortified chickpea, MAFLD, lipid accumulation, zinc, selenium

## Abstract

MAFLD progression is closely linked to a systemic failure of antioxidant defense systems. Se and Zn play crucial roles in maintaining redox balance in the liver. This study evaluated the effects of micronutrient-biofortified chickpea flours as functional ingredients for the prevention and management of MAFLD disturbances. Chickpea seeds were germinated with Na_2_SeO_3_, ZnSO_4_, ZnSeO_3_, or ZnSO_4_ + Na_2_SeO_3_, processed into flours, and then subjected to gastrointestinal digestion to obtain biofortified-chickpea digests (BCD). SDS-PAGE and FTIR indicated treatment-dependent changes in the protein/peptide profile and in the structural organization of the digested matrix. Isoflavone content was higher in ZnSO_4_-BCDs. The oleic acid-induced HepG2 cell model was used to emulate MAFLD conditions. Under preventive conditions, except for ZnSeO_3_-BCD, all treatments reduce triglyceride accumulation from 17.1 to 38.6%. Non-biofortified (GC) chickpea flour and ZnSeO_3_-BCD had greater effects on lipolysis and glycerol release. Overall, Se-BCD affected redox regulation 1.2–1.3-fold, suggesting potential improvement in lipid utilization. GC and ZnSO_4_ + Na_2_SeO_3_ BCDs decreased triglyceride accumulation (21.1 and 20.5%, respectively) when evaluated post lipid exposure. In both experimental conditions, BCDs significantly reduced IL-6 levels by 25.1 to 34.7%, demonstrating their immunomodulatory potential. Biofortified chickpea flours exhibit complementary and coordinated biological activities against the main metabolic disturbances associated with MAFLD. Zn/Se-biofortification of chickpea is a valuable strategy for addressing micronutrient deficiencies and for producing functional ingredients to prevent or ameliorate MAFLD-associated disturbances and improve liver health.

## 1. Introduction

Around the world, obesity and overweight status represent a hazard for human health, as they commonly lead to more severe non-communicable diseases. It is estimated that around 37.0% of the world population suffers from overweight status/obesity, and increased income has been related to a 14% increase in developing overweight status/obesity [1]. Excessive adipogenesis and adipose tissue expansion promote chronic, low-grade inflammation and an oxidative cellular environment, triggering systemic metabolic disturbances such as insulin resistance, dyslipidemia, central obesity, and hypertension. The clustering of these interrelated abnormalities leads to the development of metabolic syndrome [2,3]. Metabolic syndrome and its interconnected comorbidities create a persistent metabolic and inflammatory state that promotes the initiation and progression of metabolic dysfunction-associated fatty liver disease (MAFLD), identifying the liver as a primary target organ of systemic metabolic dysfunction. In prior classifications grouped within non-alcoholic fatty liver disease (NAFLD), MAFLD is characterized by hepatic steatosis in more than 5% of hepatocytes [4], differing from NAFLD by demonstrating the presence of metabolic-syndrome-related comorbidities [5]. In 2023, the term metabolic dysfunction-associated steatotic liver disease (MASLD) was introduced to establish a clearer baseline for clinical trials, thereby broadening inclusion criteria when compared to MAFLD. MASLD is better for the diagnosis of lean individuals, whereas MAFLD places greater emphasis on the association with overweight status or obesity [6,7]. The term MAFLD, however, offers a better multifactorial assessment of mortality, focusing heavily on hepatic and extrahepatic metrics that are strongly associated with hepatic fibrosis [8]. Patients with MAFLD without any lifestyle changes and medical intervention can progress into metabolic-associated steatohepatitis (MASH), with a 20–30% likelihood. Consequently, 2–5% of these patients will progress to cirrhosis [9], whereas 3–6% will develop hepatocellular carcinoma [10].

The progression of MAFLD is closely linked to a systemic failure of antioxidant defense systems, driven by the accumulation of free fatty acids that overwhelm mitochondrial oxidative capacity [11]. This redox imbalance is exacerbated by decreased activity of key antioxidant enzymes, such as glutathione peroxidase (GPx) and superoxide dismutase (SOD), both of which are strictly dependent on the availability of selenium (Se) and zinc (Zn) as essential cofactors. Both Se and Zn play crucial roles in maintaining redox balance in the liver and hepatocyte function. Moreover, specific micronutrient deficiencies such as zinc (Zn) and selenium (Se) have been associated with the prevalence of metabolic disturbances [12]. Zn and Se are both indispensable regulators of redox homeostasis and inflammatory signaling in the human body [13,14]. Zn is a key catalyst for enzymes related to DNA synthesis, RNA transcription, and immune cell functionality [15]. Similarly, Zn is an essential cofactor for superoxide dismutase (Cu/Zn-SOD) and various metallothioneins; it protects sulfhydryl groups of proteins from oxidation and stabilizes the plasma membrane against free radical damage [16]. Furthermore, adequate zinc levels inhibit pro-fibrogenic signaling pathways and improve insulin sensitivity, a key etiological factor in MAFLD, and reduce chronic low-grade inflammation [17,18]. On the other hand, Se exerts its biological effects primarily through selenoproteins such as glutathione peroxidases and thioredoxin reductases, which defend against oxidative injury [19]. Selenium deficiency compromises cell membrane integrity and promotes lipotoxicity [20].

Human studies have demonstrated that 30 mg of elemental Zn for three months improved metabolic syndrome-related markers and oxidative biomarkers in patients with MAFLD [21]. On the other hand, Se has been reported to modulate lipid metabolism and stress biomarkers associated with MAFLD in rats with induced fatty liver disease [22,23]. A combination of Se and Zn increased the resting metabolic rate of overweight patients [24], demonstrating a potential to alleviate metabolic syndrome.

Although Se and Zn are essential micronutrients, nearly two billion people worldwide have serious deficiencies, either due to low intake or low bioavailability [25,26]. In this context, diet stands out as a valuable strategy for primary prevention and management of MAFLD through the intake of essential, highly bioavailable nutrients. Biofortification of legumes and grains represents an economically and sustainably strategic approach to developing more nutritious food, not only by enriching their micronutrient content but also by improving their bioavailability [27,28,29]. In plant systems, exposure to Se/Zn has been reported to upregulate the phenylpropanoid pathway, resulting in a significant increase in phenolic content [30]. Altogether, biofortification with Se and Zn aims to go beyond nutrient density by improving a crop’s phytochemical profile and antioxidant capacity of a crop, thereby creating high-value functional foods and addressing chronic disease prevention and alleviation. For instance, chickpea is a highly nutritious matrix and an excellent candidate for biofortification, since it contains 17–22% of protein [31], along with higher dietary fiber content (18–22%) and an interesting isoflavone profile. Moreover, previous studies by our research group have demonstrated that Se-biofortified chickpea exhibits hypolipidemic, antioxidant, anti-adipogenic, and anti-inflammatory properties [32,33,34,35].

Even though Se/Zn biofortification in chickpea has been previously described by our research group, with relevant results in bioaccessibility, anti-inflammatory, and antioxidant activity [32], the study did not analyze post-digestion structural changes in protein, or the biological activity derived thereof. Taken together, this study aimed to evaluate the effects of biofortification of chickpea flours with zinc (Zn), selenium (Se), and their combination, as potential food ingredients for the prevention and amelioration of hepatic lipid accumulation and metabolism, and biomarkers of oxidative stress and inflammation associated with MAFLD, using oleic acid (OA)-induced human hepatic (HepG2) cells.

## 2. Materials and Methods

### 2.1. Biological Materials

#### 2.1.1. Cell Line

Human hepatocellular carcinoma (HepG2) cells were purchased from American Type Culture Collection (ATCC, Manassas, VA, USA).

#### 2.1.2. Chickpea Seeds

Chickpea (*Cicer arietinum* L.) seeds of the Kabuli cultivar Blanco Sinaloa were obtained from Angostura, Sinaloa, Mexico.

### 2.2. Chickpea Seed Germination and Biofortification

Chickpea seeds (*Cicer arietinum*) were disinfected and industrially germinated by Alimentos Lee ^®^ at 80% relative humidity and 22 °C for 48 h. Treatment combinations of mineral salts for biofortification were added as previously reported by Espriu-Corella et al. and Guardado Felix et al. [32,33] (see Supplementary Table S1, which states the specific components and quantities used for biofortification). Treatment groups consisted of chickpea seeds germinated with only water (germinated control, GC), and with Na_2_SeO_3_, ZnSO_4_, ZnSeO_3_, or ZnSO_4_ + Na_2_SeO_3_. Germinated seeds were dried for 24 h at 55 °C in a convection oven (Electrolux, Porcia, Italy), and ground using a coffee grinder (Krups, Mayenne, France). The resulting powder was sifted through a Monitox No. 80 mesh, resulting in flour with a particle size ≤ 0.18 mm [36].

### 2.3. Mineral Quantification

Se and Zn concentrations were assessed with the help of an inductively coupled plasma mass spectrometer X series 2 (ICP-MS, ThermoFisher, Waltham, MA, USA) as previously described by Espriu-Corella et al. [32].

### 2.4. In Vitro Gastrointestinal Digestion

Biofortified chickpea flour was digested following the INFOGEST in vitro gastrointestinal digestion methodology [37]. Essentially, specific salts and enzymes (see Supplementary Table S2 for an in-depth depiction of enzymes and chemicals utilized, along with the quantities, in each step of the in vitro digestion) were added to create simulated salivary fluid (SSF), simulated gastric fluid (SGF), and simulated intestinal fluid (SIF). SSF was added on a 1:1 (*w*/*w*) ratio for 2 min at pH 7.0. Afterwards, 10 mL of the oral bolus was mixed with 10 mL of the SGF for 2 h at pH 3.0 under agitation, followed by a 1:1 (*v*/*v*) digestion with 20 mL of the resulting gastric chyme and SIF, for 2 h at pH 7.0 under agitation. All steps were performed at 37 °C. Samples were frozen at −80 °C, and subsequently freeze-dried, yielding biofortified chickpea digests (BCD).

### 2.5. Degree of Hydrolysis

The degree of hydrolysis was calculated by measuring free alpha amino nitrogen (AAN), using the ninhydrin method [38,39]. Essentially, 2 mL of a 1 mg/mL sample was added to 1 mL of a 0.5% (*w*/*v*) ninhydrin solution (Amresco, Framingham, MA, USA). Samples were incubated in a water bath at 95 °C for 16 min and allowed to cool at room temperature (~20 °C) for 20 min. Subsequently, 5 mL of 0.2% potassium iodate (KIO_3_, Meyer, Mexico City, Mexico) was added. In a 96-well plate, 200 μL of the resulting solution was added in triplicate. After reading the absorbance at 570 nm, AAN (mg/g sample) was calculated as

(1) $$\mathrm{AAN}\left(\frac{\mathrm{mg}}{g\;\mathrm{sample}}\right)=\frac{\frac{{\mathrm{Abs}}_{s}\cdot 2\cdot \mathrm{DF}}{{\mathrm{Abs}}_{\mathrm{gly}}}}{\mathrm{Sample}\;\mathrm{concentration}\;\left(\frac{g}{L}\right)}$$

where A${\mathrm{bs}}_{s}$ is the absorbance of the sample, DF is the dilution factor, and ${\mathrm{Abs}}_{\mathrm{gly}}$ is the absorbance of the glycine working solution. The degree of hydrolysis was calculated as reported by Mudgil et al. [40]:

(2) $$\mathrm{DH}\left(\%\right)=\;\frac{{\mathrm{AAN}}_{h}-{\mathrm{AAN}}_{P}}{{\mathrm{AAN}}_{P}}\cdot 100$$

where ${\mathrm{AAN}}_{h}$ is the AAN $\left(\frac{\mathrm{mg}}{g\;\mathrm{sample}}\right)$ of the hydrolyzed sample and ${\mathrm{AAN}}_{P}$ is the AAN $\left(\frac{\mathrm{mg}}{g\;\mathrm{sample}}\right)$ of the raw protein.

### 2.6. Electrophoretic Profile

A 13.5% sodium dodecyl sulfate polyacrylamide gel electrophoresis (SDS-PAGE) was made with 29:1 acrylamide/bisacrylamide in a Mini-Protean^®^ Handcast System (BioRad, Hercules, CA, USA). Protein from raw flours was extracted by alkaline solubilization (pH 10) at 1:9 (*w*/*v*) [41]. After going through a 0.2 μm filter, samples were lyophilized. Protein extracts (20 μg) were injected into each well after sample preparation with Laemmli buffer (BioRad, USA) and β-mercaptoethanol (Amresco, USA). The PAGE was carried out at 150 V for 1.5 h. The gel was stained with 0.05% Coomassie Brilliant Blue G-250 (Sigma-Aldrich, St. Louis, MO, USA) in 40% methanol and 10% acetic acid.

A Tricine SDS-PAGE for low-molecular-weight proteins was performed as described by Haider et al. [42], with minor modifications. A 16% acrylamide/bisacrylamide (29:1) resolving gel with tricine was cast with the help of a Mini-Protean ^®^ Handcast System (BioRad, USA). BCD samples (40 μg) were loaded in each lane, and an Ultra-low Range Molecular Weight Marker (Sigma-Aldrich #M3546, USA) was used. Electrophoresis was performed at 150 V for 4 h. The gel was fixed in 5% glutaraldehyde solution for 30 min, followed by overnight staining with 0.03% Coomassie Brilliant Blue G-250 (Sigma-Aldrich, USA) in 10% acetic acid. Band intensities were analyzed using ImageJ (version 1.54r), as described by Smith et al. [43].

### 2.7. Isoflavone Extraction and Detection via Ultra-Performance Liquid Chromatography

The isoflavone extraction protocol was modified from Espriu-Corella et al. [32]. BCD digests (0.1 g) were weighed and mixed with 80% methanol at a 1:8 (*w*/*v*) ratio. Samples were mixed for 1 min in a vortex mixer (Labnet International, Edison, NJ, USA) and sonicated for 10 min (Branson, MI, USA). The dissolution was centrifuged at 10,000 rpm at 4 °C for 10 min in a Sorvall ST Plus Series Centrifuge (Thermo Fisher Scientific, Waltham, MA, USA). After decanting the liquid portion, the process was repeated with the remaining solids. The extracted liquid was filtered with 0.45 μm filters (Corning, Kaiserslautern, Germany) into HPLC vials.

Isoflavone detection was performed using an Ultra-Performance Liquid Chromatography (UPLC) separation unit, Photo Diode Array (PDA) detector, and Single Quadrupole Mass Spectrometer (QDa) detector (Acquity H Plus, Waters, Milford, MA, USA). An Acquity BEH C18 2.1 × 100 mm, 1.7 μm column was used at 30 °C with a flow rate of 0.4 mL/min, and chromatograms were recorded at 260 nm. Formic acid (0.1%) in water was used as phase A, and formic acid (0.1%) in 99.9% acetonitrile as phase B; the detailed mobile-phase gradient protocol is shown in Table 1. Samples were injected at 1 μL per run. The standard curves and samples were injected in triplicate, and chromatograms were recorded at 260 nm. The isoflavone concentrations were expressed as micrograms equivalents of biochanin A (Sigma Aldrich, St. Louis, MO, USA) per gram of biofortified chickpea digests (µEq/g BCD). The biochanin A detected in the methodology showed a linearity range (LR) of 3.125–50 µg/mL (y = 13450x + 11657, R^2^ = 0.999).

The QDA detector was set in positive mode at 80 V in the capillary and 30 V in the cone. Masses between 50 and 800 Da were detected. Identification was performed based on the UV spectrum and the *m*/*z* reported in the literature [33].

### 2.8. Attenuated Total Reflectance Fourier Transformed Infrared Spectroscopy (ATR-FTIR)

Digested and raw samples were analyzed by Fourier transform infrared spectrometry (FTIR). The IRTracer-100 (Shimadzu, Kyoto, Japan) was set to attenuated total reflectance (ATR) mode, and the spectral region was 400–4000 cm^−1^ [44]. In Origin Pro 10.2 (Learning Edition), the data between 800 and 1800 cm^−1^ was smoothed, and a baseline correction was applied for each functional region [45,46]. Normalized absorbances were plotted against each other for comparison.

### 2.9. Advanced Glycation End Product (AGE) Formation

The following solutions were prepared: 200 mM (pH 7.4) potassium phosphate buffer (PBS); running solution consisting of 22 mg/mL bovine serum albumin (BSA, Amresco, USA) and 550 mM D-fructose (Meyer, Mexico), filtered through a 0.22 μm filter (Corning, Germany); and samples at 11 mg/mL diluted in PBS. Samples were prepared in triplicate and consisted of 2 mL of running solution and 0.2 mL of sample, to obtain final concentrations of 1 mg/mL BCD, 20 mg/mL BSA, and 500 mM D-fructose [47]. The positive control (C+) consisted of 2.2 mL of 20 mg/mL BSA and 500 mM D-fructose; the negative control (C−), of 2.2 mL of 500 mM D-fructose; and the blank (B), of 2.2 mL of 20 mg/mL BSA. All test groups were incubated anaerobically for 5 days at 37 °C; this was followed by precipitation with 10% trichloroacetic acid (TCA) at a 1:1 (*v*/*v*) ratio. After recovery via centrifugation (10,000× *g*, 10 min), the precipitate was washed twice with 5% TCA. The resulting pellet was resolved in 1 mL PBS, and 200 μL were transferred to 96-well plate wells. Fluorescence was measured at 350/425 nm (excitation/emission). AGE formation was expressed as a percentage relative to the positive control.

### 2.10. Evaluation of Biologic Activities of Biofortified Chickpea Digests (BCD)

#### 2.10.1. Preparation of Oleic Acid–Bovine Serum Albumin (OA–BSA) Complex

A 12 mM oleic acid (OA) with 11% (*w*/*v*) bovine serum albumin (BSA) was prepared by diluting OA (Sigma-Aldrich, USA) in sterile PBS, followed by the addition of lyophilized BSA (Amresco, USA). The OA–BSA mixture was vortexed for 1 min and sonicated for 10 min (Branson, USA), yielding a clear, homogeneous solution. Afterwards, the solution was filtered through a 0.22 μm filter (Corning, Germany) and collected in a sterile tube for further use.

#### 2.10.2. Cell Culture

Human hepatocellular carcinoma (HepG2) cells (American Type Culture Collection, USA) were seeded in 96-well plates (1 × 10^4^ cells/well) and grown in Dulbecco’s Modified Eagle Medium-F12 (DMEM-F12) supplemented with 5% fetal bovine serum (FBS) and 1% penicillin–streptomycin (Gibco, Grand Island, NY, USA) at 37 °C and 5% CO_2_.

#### 2.10.3. Cell Viability Assay

Cell viability was determined using the CellTiter 96 Aqueous One Solution Cell Proliferation Assay (Promega, Madison, WI, USA) after treating cells under the preventive or ameliorative conditions described below. Supplementary Figure S1 represents the experimental conditions and timing for preventive and ameliorative testing in HepG2 cells.

Preventive effects: cells were treated with BCD samples (germinated control, Na_2_SeO_3_, ZnSO_4_, ZnSeO_3_, or ZnSO_4_ + Na_2_SeO_3_) at a final concentration of 40 μg/mL (for preliminary studies, see Supplementary Figures S2 and S3) for 24 h. After that, 100 µL of 0.3 mM oleic acid (OA) + 0.275% bovine serum albumin (BSA) and 40 μg/mL of BCD samples were added. The cells were incubated for an additional 24 h.

Ameliorative effects: cells were treated with 100 μL of 0.3 mM OA + 0.275% BSA for 24 h, followed by 100 μL of medium containing BCD samples (at a final concentration of 40 µg/mL) and 0.3 mM OA + 0.275% BSA for 24 additional hours.

After each experimental condition, cells were treated with 10 μL of CellTiter 96 Aqueous One Solution Cell Proliferation Assay and incubated at 37 °C for 45 min. Absorbance was measured at 490 nm with a microplate reader (Synergy HT, Bio-Tek, Winooski, VT, USA).

#### 2.10.4. Lipid Accumulation in HepG2 Cells Assessed via Oil Red O (ORO) Staining

Cells were seeded and treated as described in Section 2.10.3. After that, and following the methodology described by Serrano-Sandoval et al. [48], with minor modifications, cells were fixed with 4% paraformaldehyde for 1 h at room temperature and permeabilized with 60% isopropyl alcohol for 60 s. After washing with phosphate-buffered saline (PBS), 100 μL of ORO (Oil Red O, Fluka Chemicals, Feltham, UK) working solution was added, and the sample was incubated for 20 min at room temperature. The cells were washed 4 times with 100 μL of distilled water, and then 100 μL of isopropyl alcohol was added to dissolve the internalized ORO. The absorbance was measured at 510 nm with a microplate reader (Synergy HT, Bio-Tek, USA). Lipid accumulation was expressed as a percentage relative to the positive control (C+).

Preparation of ORO working solution was as follows: 250 mg of ORO powder was added to 40 mL of 100% isopropyl alcohol and incubated statically at 56 °C for 24 h. Afterwards, the mixture was gauged at 50 mL, and filtered through a Whatman 1 filter, yielding the ORO stock solution. Two hours before use, the stock solution must be incubated at 60 °C. The ORO working solution consists of 3 parts stock solution and 2 parts distilled water, and must be filtered through a 0.22 μm membrane.

#### 2.10.5. Triglyceride and Glycerol Quantification

The concentration of triglycerides accumulated in cells was quantified using Abcam’s Fluorometric Triglyceride Quantification Assay Kit (ab65336, Cambridge, MA, USA), following the manufacturer’s instructions. Triglyceride concentration was calculated as nmol/mL and expressed as a percentage relative to the positive control (C+). Likewise, the glycerol release was measured using Abcam’s Cell-Based Glycerol Assay Kit (ab133130, Cambridge, MA, USA) and according to the instruction manual. Glycerol concentration was calculated as free glycerol (μg/mL) and expressed as a percentage relative to the positive control (C+).

#### 2.10.6. Interleukin 6 (IL-6) Release

Interleukin (IL)-6 concentration was determined in the supernatants recovered from both experimental conditions, protective and ameliorative, using a Quantikine ELISA Human IL-6 Immunoassay (D6050, R&D Systems, Minneapolis, MN, USA) according to the manufacturer’s instructions. In biological duplicates, IL-6 released from cells was calculated in pg/mL and expressed as a percentage relative to the positive control (C+).

#### 2.10.7. Glutathione Peroxidase (GPx) Activity

Glutathione peroxidase activity was determined with a Glutathione Peroxidase Assay Kit (703102, Cayman Chemicals, Ann Arbor, MI, USA). Preventive and ameliorative efforts were evaluated, according to the manufacturer’s instructions. GPx activity was expressed as a percentage relative to the negative control (C−), which served as an unaltered antioxidant control, allowing for comparisons for up- and downregulated activity.

### 2.11. Statistical Analysis

At least three independent experiments were performed for each sample, except for specific kits, which were performed in duplicate, as per the manufacturer’s instructions or recommendations. Results are expressed as mean ± standard deviation (SD). The JMP (Pro 14) software (SAS Institute Inc., Cary, NC, USA) was used to perform all statistical analyses. After performing a one-way ANOVA (*p* < 0.05), Tukey’s HSD post hoc test was used to compare treatments at a 95% confidence level. When pertinent, a two-sample *t*-test was performed to detect statistical differences between treatments, and Tukey’s HSD post hoc test was used to test treatment differences.

## 3. Results and Discussion

### 3.1. Structural Characterization of Biofortified Chickpea Digests (BCD)

#### 3.1.1. Zn and Se Quantification and Bioaccessibility

As shown in Supplementary Figure S4A, which states the amounts of Zn and Se present in each digested sample, Zn was significantly more abundant across all treatments. Zn uptake increased with Zn biofortification, but was even higher in the presence of Se, even without Zn addition. The Zn quantification, in decreasing order, goes as follows: ZnSO_4_ + Na_2_SeO_3_ > ZnSeO_3_ > Na_2_SeO_3_ > ZnSO_4_ > GC, without a statistical difference between ZnSeO_3_ and Na_2_SeO_3._ This behavior has been previously observed in mung bean (*Vigna radiata* L.), demonstrating that Zn and Se share overlapping transcriptomic processes related to mineral intake, sulfate transporters, and various enzymatic reactions [49]. Furthermore, germination alone is responsible for the breakdown of phytic acid, which in turn promotes the release of Zn, otherwise chelated by this acid [50,51,52]. Differently, Se was more abundant in samples supplemented with Na_2_SeO_3_ and ZnSO_4_ + Na_2_SeO_3_, followed by ZnSeO_3_. In terms of Se abundance, ZnSO_4_ behaved similarly to the germinated control. Likewise, Se bioaccessibility in GC was statistically higher (~148%) than this value in any of the other biofortified chickpea flours (Supplementary Figure S4B). No differences in Se bioaccessibility were observed between biofortified chickpea flours. Similar behavior in Se bioaccessibility was previously reported by Espriu-Corella et al. [32]. It has been reported that Se has a narrow margin of safety, so dose control is very important. Based on bioaccessibility results, consumption of ~11–13 g of Se-biofortified chickpea flour (5.12–5.16 μg Se/g flour, dry weight) can meet the daily recommendations for adults (55–70 µg Se/day) [53]. These amounts of biofortified chickpea flour are much lower than those that would be equivalent to maximum intake levels of Se (255–400 µg Se/day in adults, equivalent to 50–80 g of biofortified flour/day) [54]. Regarding Zn bioaccessibility, only chickpea flour biofortified with ZnSO_4_ showed statistically higher Zn bioaccessibility than GC (*p* < 0.0195). The observed trends point out that individual biofortification with ZnSO_4_ or Na_2_SeO_3_ favored bioaccessibility of Zn.

#### 3.1.2. Protein Profile

The alpha-amino nitrogen (AAN) assay was used to determine the degree of protein hydrolysis (DH) in the BCD samples (Supplementary Table S4 reports AAN quantification for each sample, which is used to calculate DH). The statistical analysis revealed that the sample biofortified with ZnSeO_3_ had a statistically higher (*p* < 0.05) DH (65.61 ± 1.60%), whereas samples with ZnSO_4_ behaved similarly to the germinated control (GC) (41.86 ± 0.50% and 45.01 ± 3.37%, respectively). In contrast, the samples severally biofortified with Na_2_SeO_3_ and ZnSO_4_ + Na_2_SeO_3_ had DH values of 12.27% and 19.67%, respectively, which were lower than the GC.

**Figure 1:**
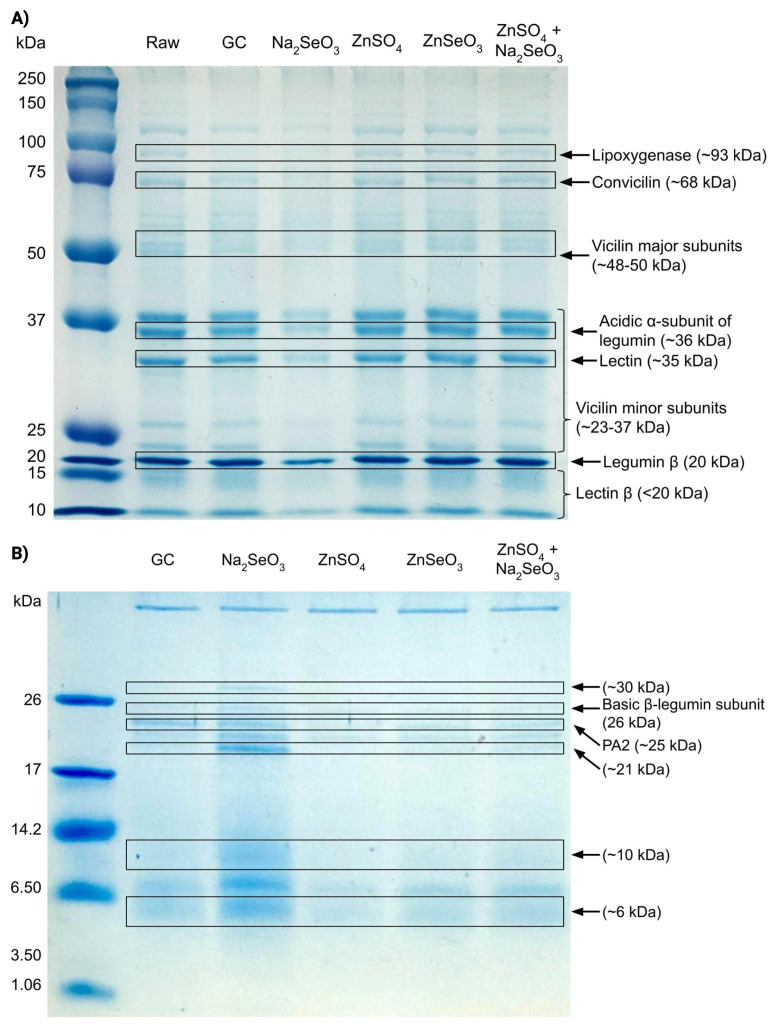
Electrophoretic profile of (**A**) biofortified chickpea flours via SDS-PAGE, and (**B**) biofortified chickpea flour digests via low molecular-weight tricine-SDS-PAGE. Raw = non-germinated control, GC = germinated control.

The SDS-PAGE of non-digested proteins (Figure 1A) showed clear differences among the non-germinated control (Raw), germinated control (GC), and biofortified samples. Based on previous proteomic characterizations of biofortified chickpea [55,56], multiple bands hypothesized to be vicilin major subunits are observed at ~50 kDa, along with convicilin at 68 kDa. Similarly, a band at (~35 kDa) has been previously reported in chickpea as lectin [55]. A substantial reduction in the expression of lectin bands was observed in the GC (85.4%) and Na_2_SeO_3_ (50.8%) samples compared to the non-germinated sample (Raw), suggesting conformational changes during germination. Likewise, biofortification with Na_2_SeO_3_ reduced the expression of a 20 kDa band by 46.8% compared with the GC sample, which was inferred to be β-legumin, potentially confirming that Se enhances germination by improving nitrogen and amino acid utilization. Based on a *Medicago sativa* L. [57] study, lipoxygenase can be found at ~93 kDa, which might be similarly expected in chickpea. An increase in lipoxygenase was observed in the ZnSO_4_, ZnSeO_3_, and ZnSO_4_ + Na_2_SeO_3_ samples, which could indicate enhanced activation of lipid metabolism and a stress-responsive pathway. Lastly, other vicilin minor subunits (>20, <37 kDa), as previously characterized in sprouted chickpea [55,56,58], appear less intense but follow a similar relative-abundance pattern. Full proteomic analysis is recommended to definitively validate these functional implications.

After gastrointestinal digestion, biofortified chickpea digests (BCD) showed low molecular-weight bands ranging from ~6 to 30 kDa (Figure 1B). In contrast to the non-digested profile, Na_2_SeO_3_ had the highest overall relative abundance of lower-molecular-weight protein fragments. Five bands were observed between ~17 kDa and ~30 kDa, some of which may correspond to vicilin and legumin subunits, storage proteins that have been shown to resist simulated digestion [59]. A 25 kDa band was observed in the GC and Na_2_SeO_3_ samples, which potentially corresponds to chickpea albumin (PA2), a functional protein that has also been reported to be resistant to simulated digestion [60]. Likewise, an intense ~21 kDa band was observed in the Na_2_SeO_3_ sample, consistent with the findings of Wang et al. [61], who demonstrated that the presence of Se leads to strong binding of protein subunits. Also, it has been suggested that Se confers greater protein stability by interacting with S-containing amino acids, limiting their degradation during gastrointestinal digestion [62]. Two diffuse bands are observed at around 10 kDa, and are expressed 50% more in Na_2_SeO_3_ than in GC, whereas the rest of the samples show a significant decrease (Na_2_SeO_3_ > GC > ZnSO_4_ + Na_2_SeO_3_ > ZnSeO_3_ > ZnSO_4_). Lastly, two large bands at ~5 and ~7 kDa are observed, suggesting the presence of multiple protein fragments with similar molecular weights. Compared to the control (GC), chickpea biofortification with Na_2_SeO_3_ increased the relative abundance of these protein fragments by 60.4%. In contrast, biofortification with ZnSO_4_ reduced the abundance of these protein fragments by 30%, relative to the GC. The presence of these low-molecular-weight protein fragments could be linked to the enhanced biological activity of the samples [63,64,65].

#### 3.1.3. ATR-FTIR

**Figure 2:**
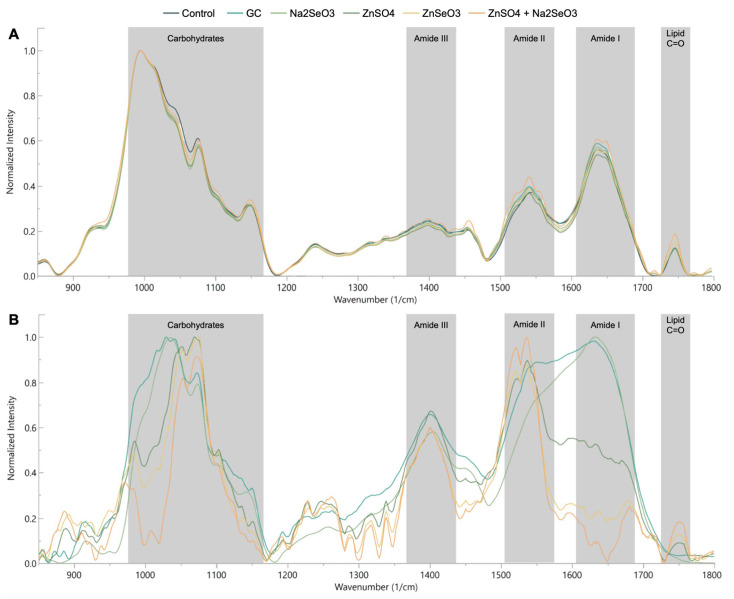
Attenuated total reflectance Fourier transformed infrared spectroscopy (FTIR-ATR) of (**A**) biofortified chickpea flours and (**B**) biofortified chickpea digests, with normalized intensity, highlighting zones of interest: Amide I (~1650 cm^−1^), Amide II (~1550 cm^−1^), Amide III (~1400 cm^−1^), Carbohydrates (980–1200 cm^−1^), and lipid C=O (1710–1760 cm^−1^). GC = germinated control.

After smoothing and baseline correction, regions of interest were delimited as previously reported in various matrices, including chickpea [46,66,67,68]. FTIR-ATR spectra indicated that germination and mineral biofortification induced mineral-specific protein remodeling without disrupting the overall macromolecular framework of chickpea (Figure 2A). In flours, moderate increases in amide I (~1650 cm^−1^) and amide II (~1550 cm^−1^) intensities, particularly in GC and Na_2_SeO_3_ + ZnSO_4_, reflected germination-driven protein restructuring [69,70], and are likely associated with reserve protein mobilization. Concurrently, a decrease in intensity near ~1050 cm^−1^ suggested partial starch disorganization, consistent with early enzymatic softening of the carbohydrate matrix.

After simulated gastrointestinal digestion (Figure 2B), differences among treatments became more apparent. The GC digest retained distinct amide I and II bands, indicating the presence of structured peptide fragments after digestion [71]. Se-biofortified samples showed changes primarily in the amide II region, with limited displacement of the amide I band. These spectral variations may reflect changes in the local environment of peptide bonds, possibly associated with alterations in hydrogen bonding, peptide exposure, or protein/peptide organization after digestion. However, these observations should be interpreted cautiously, as FTIR does not provide direct evidence of specific Se-protein binding mechanisms [72,73]. In ZnSO_4_-biofortified samples, changes in the amide I and amide II regions, along with variations in the carbohydrate-associated region, suggest that Zn biofortification influenced the structural organization of the digested matrix. These changes are consistent with alterations in protein/peptide conformation and in the interaction environment among peptides, minerals, carbohydrates, and other digested components. Nevertheless, FTIR alone cannot confirm Zn coordination chemistry or the formation of defined Zn-protein domains. Therefore, the spectral changes observed here should be considered indicative of matrix-level structural modifications rather than direct evidence of specific coordination mechanisms [74]. In samples containing both Zn and Se, the FTIR profiles showed intermediate or treatment-specific spectral patterns, suggesting that the combined biofortification strategy altered the organization of the digested chickpea matrix differently than the individual mineral treatments [69,75,76].

These changes may reflect the combined effects of mineral availability, peptide profiles, and bioactive compounds generated or released during germination and gastrointestinal digestion. Additionally, the increase in the lipid carbonyl region around ~1740–1745 cm^−1^ may indicate greater exposure of ester-containing compounds after digestion. The FTIR results support the presence of treatment-dependent structural differences in biofortified chickpea flours and digests. However, the specific molecular interactions among minerals, peptides, and polyphenols should be considered tentative and require complementary analytical approaches for confirmation.

#### 3.1.4. Isoflavone Quantification Using UPLC-PDA-QDa

Malonylated formononetin glycoside (MFG), formononetin, and biochanin A were the isoflavones detected and quantified in biofortified chickpea digests (BCD) (Table 2). Biofortification increased the total content of isoflavones in chickpea digests in the following order: ZnSO_4_ > Na_2_SeO_3_ > ZnSeO_3_ = Na_2_SeO_3_ + ZnSO_4_ > GC. Compared with GC, the total isoflavone content increased by 84.5% when chickpea seeds were biofortified with ZnSO_4_, whereas it increased by 49.4% with Na_2_SeO_3_. Se and Zn modulate plant secondary metabolism, thereby acting as elicitors of bioactive-compound synthesis. Evidence suggests that Se and Zn increase total phenolic and flavonoid contents through upregulation of the phenylpropanoid pathway [30].

A similar tendency was observed by Limón-Aguilera et al. [77], who reported increases in the total isoflavone content from chickpea digest of 143.7% and 75.2% when the chickpeas were biofortified with ZnSO_4_ and Na_2_SeO_3_, respectively. This behavior can be explained by the increased activity of key enzymes, such as 3-deoxy-D-arabino-heptulosonate-7-phosphate (DAHP) synthase and the phenylalanine ammonia-lyase (PAL), which are strongly involved in the biosynthesis of phenolic compounds in response to stress [32,78,79]. As expected, aglycone forms formononetin and biochanin A were the most abundant in all samples because of the breakdown of the glycosidic and other conjugated linkages during gastrointestinal digestion [80].

Proteins and peptides can form stable, non-covalent complexes with multiple phenolic compounds, primarily due to hydrophobic interactions. These interactions induce changes in secondary structures as binding promotes conformational remodeling [81,82,83,84], which, in turn, protects polyphenols from degradation and oxidation. The protective effect of protein–polyphenol complexes can be seen in the availability of compounds post-digestion [32], where, compared to undigested biofortified chickpea flours, malonylated formononetin glycoside is 70.9% more accessible in the germinated control. In contrast, when compared to undigested samples, Zn and/or Se biofortification could have promoted the creation of peptide–polyphenol structures, which are able to increase the post-digestion accessibility of formononetin and biochanin A by 2.08–3.48-fold and 1.78–4.01-fold, respectively. The germinated control showed no improvement in accessibility in either case. This demonstrates how polyphenols liberated through enzymatic digestion can remain protected and accessible in BCDs.

#### 3.1.5. Advanced Glycation End-Product (AGE) Formation

As a condition related to metabolic syndrome, MAFLD is similarly driven by oxidative stress, insulin resistance, and carbonyl stress, including the generation of highly reactive dicarbonyl intermediates, such as methylglyoxal (MGO), which form AGEs [85]. Moreover, the progression of steatosis into steatohepatitis and hepatic fibrosis relies on the activation of hepatic stellate cells (HSCs), which express the AGE receptors (RAGE). When bound to AGEs, reactive oxygen species (ROS) are released, and pro-fibrotic signaling cascades are triggered [86,87]. Thus, inhibition of in vitro AGE formation suggests the potential to diminish metabolic stress on hepatic cells.

**Figure 3:**
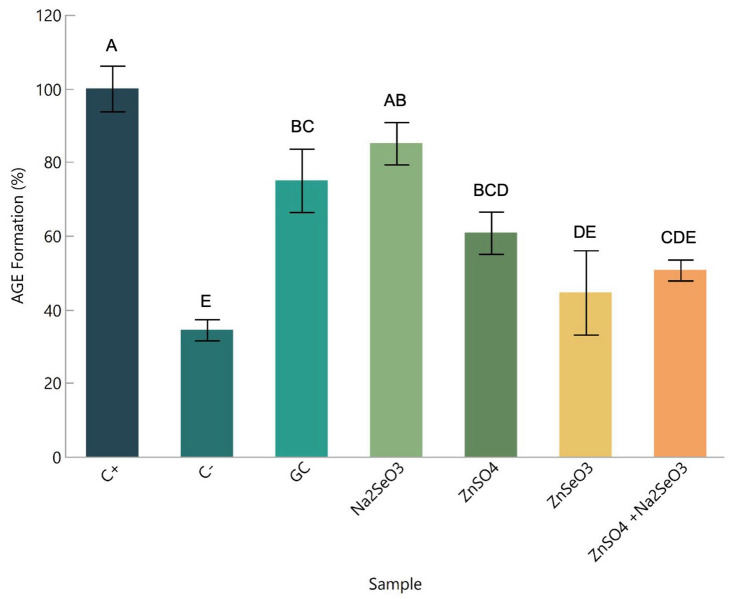
Advanced glycation end-product (AGE) formation when treated with biofortified chickpea digests, compared to the positive control (C+). C− = negative control, GC = germinated control. ^A–E^ Different letters indicate statistically significant differences (*p* < 0.05) amongst treatments in a Tukey’s HSD post hoc test (n = 3).

Compared with the positive control (not containing BCDs), all samples decreased (*p* < 0.05) AGE formation, except for Na_2_SeO_3_, which, despite showing a tendency to decrease AGE formation, did not demonstrate a statistical difference (Figure 3). BCDs with ZnSeO_3_ and ZnSO_4_ + Na_2_SeO_3_ reduced AGE formation by 55.41% and 49.32%, respectively, values very similar to those of the negative control (C−). Overall, the three Zn-supplemented samples tend to reduce AGE formation, suggesting a potential benefit in reducing glycation- and oxidation-mediated afflictions in patients with metabolic syndrome.

As an adduct formation between the dicarbonyl groups of proteins and reducing sugars, AGE formation has been implicated in several metabolic syndrome-related diseases. AGEs, formed under hyperglycemia and hyperlipidemia, or otherwise ingested from high-AGE Western diets, bind to the receptor for AGEs (RAGE) on adipocytes, macrophages, and endothelial cells, activating NF-κB and MAPK signaling, increasing reactive oxygen species, and driving low-grade inflammation, insulin resistance, and endothelial dysfunction [88,89]. Zn has consistently demonstrated attenuation of AGE formation and its downstream toxicity in vitro and in vivo models. This activity has most likely been attributed to the limitation of oxidative and carbonyl stress and to the preservation of nitric oxide bioavailability [90]. Furthermore, Zn-enriched spirulina was shown to suppress AGE-RAGE-NF-κB signaling in diet-induced obese mice, thereby reducing the AGE burden [91]. These findings highlight the multimodal anti-glycation role of zinc, which acts at two levels, namely, AGE formation and interference with signaling, both of which perpetuate glycoxidative damage.

The antiglycation potential of trace minerals was previously studied by Khan et al. [92]. It was demonstrated that a dose–response effect occurred with the addition of Zn in reducing AGE formation, as observed through fluorescent spectroscopy. Considering the increased presence of isoflavones (a type of polyphenol) when compared to the germinated control (Table 2), a decrease in AGE formation can be expected. Similarly, the role of trace elements (Se/Zn) in inhibiting glycation has been previously described. While both trace elements decrease oxidative stress and support antioxidant defenses and immunity [93], the mechanisms by which this is achieved vary; selenium is known to decrease glycation sites [94,95], whereas zinc reduces the binding of protein residue to reducing sugars [96,97].

### 3.2. Biological Potential of Biofortified Chickpea Digests (BCD)

#### 3.2.1. Effects of BCD as a Prevention Strategy in In Vitro HepG2 Hepatic Steatosis Models

##### Lipid Accumulation and Metabolism

**Figure 4:**
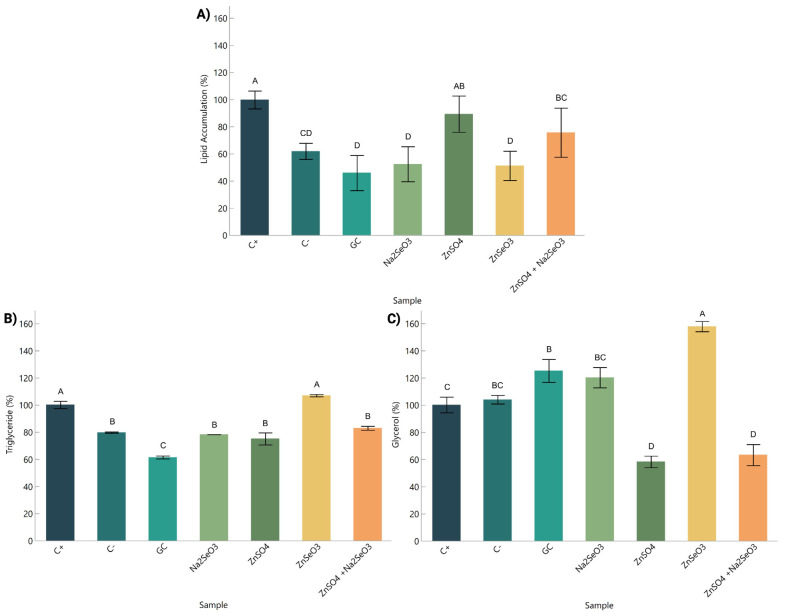
Preventive effects of biofortified chickpea digests (BCD) on lipid metabolism of HepG2 cells under hepatic steatosis conditions: (**A**) lipid accumulation by Oil Red O (ORO) staining (n = 3), (**B**) triglyceride accumulation (n = 2), and (**C**) glycerol release (n = 3). C+: positive control treated with OA but without BCD, C−: negative control without OA or BCD. ^A–D^ Different letters indicate statistically significant differences (*p* < 0.05) amongst treatments in a Tukey’s HSD post hoc test.

All treatments and concentrations described herein resulted in a cell viability of 90% or more, as seen in Supplementary Figure S2. Compared to the positive control (C+), lipid accumulation in HepG2 cells was significantly reduced (*p* < 0.05) by the germinated control (GC) and by biofortified chickpea digests (BCD) containing ZnSeO_3_ and Na_2_SeO_3_, to levels similar to the negative control (Figure 4A). In contrast, cells treated with ZnSO_4_ did not differ from the positive control, demonstrating a lack of efficacy in preventing lipid accumulation. Centrone et al. [98] reported a positive effect of chickpea extracts on rat hepatoma (FaO) cells, reducing lipid accumulation as a preventive strategy. Furthermore, the roles of zinc [99] and selenium [100] in preventive strategies to inhibit lipid deposition have been previously reported, highlighting their potential at supranutritional levels.

Lipid droplet accumulation in hepatic cells, a key characteristic of metabolic dysfunction-associated fatty liver disease (MAFLD), primarily consists of triglyceride accumulation [101,102]. Under normal conditions, cells must maintain lipid homeostasis, with lipid uptake and excretion balanced. After induction with oleic acid (OA), cells treated with germinated control (GC) and Na_2_SeO_3_ significantly (*p* < 0.05) decreased triglyceride (TG) accumulation (Figure 4B) by 17.1–38.6% and showed a tendency to increase glycerol release (Figure 4C), despite showing no statistical difference (*p* < 0.05) when compared to the positive control (C+). In contrast, ZnSeO_3_ did not show a decrease in triglyceride accumulation compared to C+ but showed the greatest increase in glycerol release (157.6%). Although triglyceride accumulation did not decrease, as lipid accumulation decreased and glycerol release increased, an enhanced energy balance in HepG2 cells could result from augmented lipolytic activity [103].

Even though ZnSO_4_ and ZnSO_4_ + Na_2_SeO_3_ decreased triglyceride accumulation, their glycerol release was lower than that of the positive control. Instead of promoting lipolysis, ZnSO_4_ could hinder triglyceride synthesis mechanisms [104]. In contrast, supplementation with Zn can promote a shift in hepatocyte phenotype towards a metabolically active, lipid-utilizing phenotype. Potential mechanisms by which Zn supplementation in hepatocytes could act include enhanced fatty acid β-oxidation and lipogenesis suppression [99]. Similarly, autophagy-mediated lipophagy and lipolysis could have been activated in hepatocytes via the Zn^2+^/MTF-1/PPARα pathway [105]; pertinent analyses would confirm said mechanisms.

##### IL-6 Release

**Figure 5:**
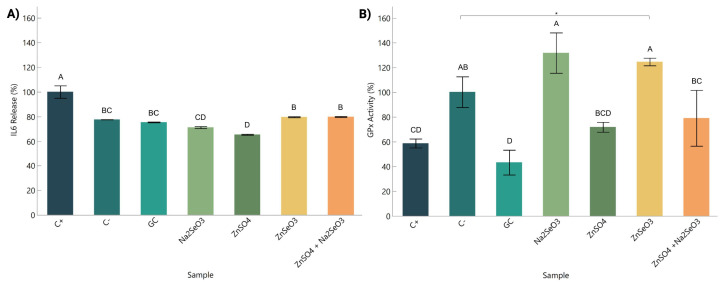
Preventive effects of biofortified chickpea digests (BCD) on oxidative stress in HepG2 cells under hepatic steatosis conditions: (**A**) interleukin 6 (IL-6) release (n = 2) and (**B**) glutathione peroxidase (GPx) activity (n = 3). C+: positive control treated with OA but without BCD, C−: negative control without OA or BCD. ^A–D^ Different letters indicate statistically significant differences (*p* < 0.05) amongst treatments in a Tukey’s HSD post hoc test. * Statistically significant differences (*p* < 0.05) between samples in a two-sample *t*-test.

As shown in Figure 5A, all treatments significantly decreased (*p* < 0.05) IL-6 production in HepG2 cells, compared to the positive control (C+). Cells treated with ZnSO_4_ reduced IL-6 release by 34.72%, followed by Na_2_SeO_3_ (28.87%), reaching levels below the untreated negative control. No significant differences (*p* > 0.05) were observed between ZnSO_4_ + Na_2_SeO_3_ or Na_2_SeO_3_ and ZnSO_4_. Under a steatotic burden, a chronic inflammatory response is triggered, in which inflammatory cytokines are released [15]. Zn has been reported to decrease IL-6 production by inhibiting the NF-κB pathway [106,107] and to ameliorate oxidative stress in liver cells, thereby decreasing the inflammatory response [16,108]. Similarly, phenolic compounds from beans have been shown to inhibit the same pathway, resulting in a decrease in pro-inflammatory cytokine release [109]. Isoflavones from legumes, which are like the ones present in the samples, have been associated with the activation of the anti-inflammatory transcription factor Nrf2 [110,111,112] and maintaining homeostasis in liver cell’s oxidative environment [113]. Based on the results, we hypothesized that similar mechanisms could potentially be active in these samples.

##### Antioxidant Activity

The roles of isoflavones, peptides, and micronutrients in the amelioration of inflammatory responses has been partly attributed to their reduction of oxidative stress [114]. Among the major antioxidant enzymes in the body, glutathione peroxidase (GPx) plays a crucial role in detoxifying peroxides. Moreover, as a selenoprotein, GPx benefits greatly from Se in terms of activity [100,115]. Hao et al. [81] emphasize the improved antioxidant profile of peptide–polyphenol complexes from legumes, which results in an increased radical-scavenging activity.

Comparing GPx levels to basal levels (C−) elucidates the damage to antioxidant defenses, which is inherent for HepG2 cells when exposed to oleic acid (C+), as well as the potential recovery of GPx activity in the presence of BCDs. Basal levels of GPx (C−) were dramatically altered (41.4 ± 3.62% decrease) by the exposure to oleic acid (OA), as well as in some treatment groups (Figure 5B). Cells treated with (SeO_3_)^−2^ not only withstood the negative effects of OA but also tended to increase GPx activity. Even more so, ZnSeO_3_ demonstrated a statistically higher (*p* < 0.05) GPx activity (1.3-fold), which was consequent to the abundance of Se, which becomes integrated into selenoproteins such as GPx. Selenium is a key component of selenoproteins such as GPx; without it, this enzyme can not only be improperly synthesized but also become catalytically weak due to a lack of selenocysteine [116,117]. In HepG2 cell culture, adding 1 µM Na_2_SeO_3_ to the culture medium significantly increased GPx activity [118].

Overall, Se-biofortification modulates lipid accumulation and its associated redox state, whereas Zn-biofortification enhances modulation of the inflammatory response. The combination of both can, in some cases, promote further improvement, most likely because structural alterations that occur after in vitro digestion can alter mechanisms by which BCDs interact with cell receptors (see Figure 2B).

#### 3.2.2. Effect of BCD on Amelioration of In Vitro HepG2 Hepatic Steatosis

##### Lipid Accumulation and Metabolism

**Figure 6:**
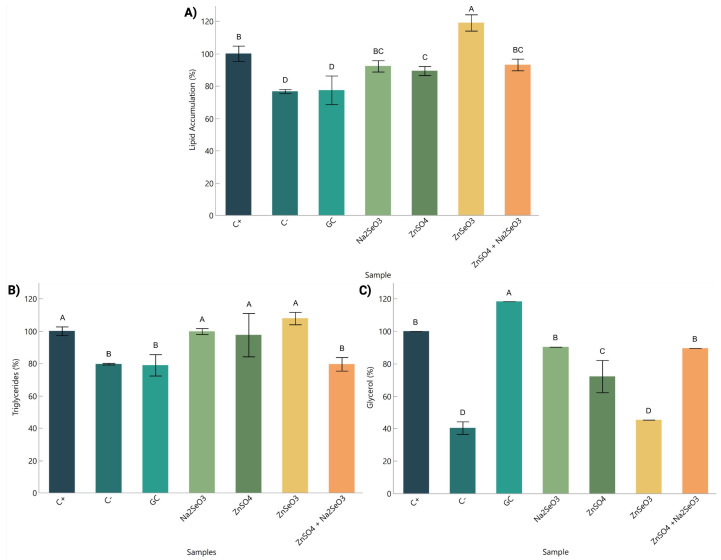
Ameliorative effects of biofortified chickpea digests (BCD) on lipid metabolism of HepG2 cells under hepatic steatosis conditions: (**A**) lipid accumulation by Oil Red O (ORO) staining (n = 3), (**B**) triglyceride accumulation (n = 2), and (**C**) glycerol release (n = 3). C+: positive control treated with OA but without BCD, C−: negative control without OA or BCD. ^A–D^ Different letters indicate statistically significant differences (*p* < 0.05) amongst treatments in a Tukey’s HSD post hoc test.

Unlike in preventive conditions, the germinated control (GC) reduced lipid accumulation to values similar to those of the negative control (C−), with no statistically significant differences (*p* > 0.05; Figure 6A). Likewise, ZnSO_4_ BCDs also reduced lipid droplet accumulation in HepG2 cells, whereas ZnSeO_3_ BCDs showed an increase in lipid accumulation (*p* < 0.05), which could be related to their higher DH and the increase in the signal at the lipid region of the FTIR analysis, suggesting a higher presence of lipids resulted from an efficient gastrointestinal digestion process (Figure 2). Dibwe et al. [101] conducted a similar study by evaluating the effect of bean extracts on lipid accumulation in HepG2 cells induced with 0.25 mM oleic acid. With concentrations ranging from 125 to 500 µg/mL, the authors demonstrated up to 100% inhibition of lipid accumulation, achieving values similar to those of the negative control in a dose-dependent manner. Moreover, chickpea flavonoids have been shown to significantly reduce lipid accumulation in hepatic metabolic models in a dose-dependent manner at 10–40 µg/mL [35].

Lipid accumulation results from imbalances between lipid breakdown and fatty acid oxidation, and between lipid uptake and lipogenesis [119], among which triglyceride accumulation and glycerol release are common indicators. Triglyceride accumulation in HepG2 cells treated with BCDs differed from that of the prevention group. While the germinated control (GC) and chickpea digests biofortified with ZnSO_4_ + Na_2_SeO_3_ decreased triglyceride accumulation by 21.1% and 20.5%, respectively, chickpea digests biofortified with Na_2_SeO_3_, ZnSO_4_, or ZnSeO_3_ did not significantly alter triglyceride accumulation in OA-induced cells (Figure 6B). The fact that the combined treatment (with ZnSO_4_ + Na_2_SeO_3_) proved to enhance further the modulatory potential of the individual treatments is explained by the complementarity of these micronutrients, as they address overlapping, but not identical, mechanisms involved in lipid-induced hepatocellular alterations, impaired lipid metabolism, oxidative stress, and inflammation [13,14,120].

The presence of selenium has been shown to improve the digestion of certain carbohydrates [121], and a surplus of it is commonly stored as lipids in the liver. In contrast, Zn supplementation has been associated with reduced triglyceride accumulation and increased B12 levels [122], factors which help to prevent lipogenesis. Nonetheless, the steatotic burden may have exceeded the capacity of the samples to promote these activities. Compared with the positive control (C+), cells treated with the germinated control (GC) showed a statistically significant (*p* < 0.05) increase in glycerol release (Figure 6C). In contrast, cells treated with ZnSO_4_ and ZnSeO_3_ released 27.9% and 54.7% less glycerol, respectively, compared to C+. These findings are consistent with those from preventive effects evaluation, where alternate lipid metabolism forms, such as increased lipophagy, decreased lipogenesis, and increased lipid β-oxidation, can result from hepatic supplementation with Zn [99,104].

Once cells are exposed to oleic acid, triglyceride synthesis and storage occur, thus establishing an accumulative phenotype resistant to reversal. Furthermore, metabolism-enhancing compounds have shown better results as preventive agents, as restoring lipid turnover and reactivating impaired metabolic pathways can take longer and be less successful [123,124]. Zhou et al. [35] demonstrated a reduction of 15.7–29.5% in intracellular triglycerides in HepG2 cells when administering chickpea flavonoids. The effect of flavonoids on lipid metabolism has been previously described by Gao et al. [125], highlighting the role of chickpea germination in increasing isoflavone activity, and thereby decreasing lipid accumulation and triglyceride content. Additionally, Wang et al. [126] have isolated the flavonoid biochanin A from chickpea and reported a 11.0% decrease in triglyceride content after oleic acid induction, findings consistent with this research. They have attributed this effect to the activation of the SIRT3/AMPK/ULK-1 autophagy pathway. The similarity with this study suggests that the same pathway might be activated.

##### IL-6 Release

**Figure 7:**
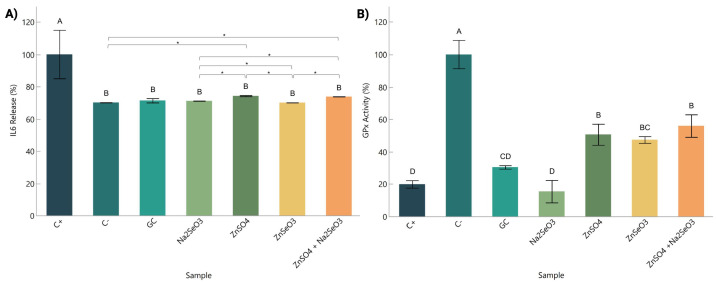
Ameliorative effects of biofortified chickpea digests (BCD) on oxidative stress in HepG2 cells under hepatic steatosis conditions: (**A**) interleukin 6 (IL-6) release (n = 2) and (**B**) glutathione peroxidase (GPx) activity (n = 3). C+: positive control treated with OA but without BCD, C−: negative control without OA or BCD. ^A−D^ Different letters indicate statistically significant differences (*p* < 0.05) amongst treatments in a Tukey’s HSD post hoc test. * Statistically significant differences (*p* < 0.05) between samples in a two-sample *t*-test.

Unlike lipid-metabolism assays, the effect of BCDs in reducing the inflammatory response was similarly successful in treatment and prevention. As seen in Figure 7A, all treatment groups significantly (*p* < 0.05) decrease IL-6 concentrations by 25.1–30.0% compared to the positive control (C+) and show significant differences between biofortified samples. The use of selenium has been reported to counteract stress-induced pathways, highlighting its relevance in maintaining an anti-inflammatory environment in human hepatic cells [127]. Selenium has been shown not only to play a potent role in attenuating lipid-induced inflammatory responses but also to reduce the progression of hepatic disease into hepatocellular carcinoma [128,129,130], a complication of untreated metabolic dysfunction-associated fatty liver disease. The combination of two salts (ZnSO_4_ + Na_2_SeO_3_) did not further decrease IL-6 production compared to the individual salts. This activity can potentially be attributed to the presence of Zn, which can modulate metallothionein, Zn transporters, and Zn-dependent enzymes, exerting an attenuated, indirect effect on IL-6 release [16,131]. Unlike Se, at higher doses (≥30–100 μM), Zn can promote IL-6 production [132,133]; thus, in this case, the treatments could approach an immunostimulatory effect, resulting in a slight increase in IL-6 release. Further testing would elucidate the mechanistic processes derived from Se/Zn exposure and the potential cooperative activity among the different components of biofortified chickpea flours.

##### Antioxidant Activity

The impaired antioxidant activity of hepatic cells in a lipid-rich environment is shown in Figure 7B. GPx activity was compared with the negative control, which was not exposed to oleic acid, allowing observation of reduced antioxidant activity and its potential amelioration. Cells severally treated with ZnSO_4_, ZnSeO_3_, and ZnSO_4_ + Na_2_SeO_3_ showed improved GPx activity that was statistically higher (*p* < 0.05) than the positive control. Unlike in the evaluation of prevention effects, samples treated with -SeO_3_ were not the primary factor behind the improvement in GPx activity, suggesting different mechanisms. The presence of polyphenol–peptide complexes can ameliorate the pro-oxidant microenvironment, as polyphenols are more readily available and resistant to degradation. In lipid systems, these conjugates confer improved oxidation resistance, highlighting their potential to prevent lipid peroxidation [84]. This activity has been attributed to the capability to bind pro-oxidant metals and reduce hydroperoxide formation [82].

In non-alcoholic fatty liver disease (NAFLD), selenium supplementation has been shown to reduce lipid peroxidation and reactive oxygen species, thereby slowing fibrosis progression [100,134] following exposure to hyper-lipidic environments. Furthermore, Goel et al. [135] concluded that zinc treatment in male rats improved hepatic histoarchitecture and normalized GPx activity after chlorpyrifos intoxication. Zn supplementation has similarly proven useful in chronic liver disease, improving antioxidant profiles by neutralizing ROS and promoting adequate levels of protein synthesis in response to inflammatory events [136,137]. These mechanisms could explain the increased GPx activity observed in samples containing Zn. Furthermore, Lv et al. [76] demonstrated the effectiveness of polypeptide–bimetallic (Zn and Se) platforms in reducing the inflammatory response and restoring redox balance by restoring mitochondrial function in hepatic cells. A similar mechanism could be expected in BCDs, as small peptides, likely carriers of Zn and/or Se, could exhibit similar activity, thereby improving antioxidant and anti-inflammatory profiles.

### 3.3. Multivariate and Correlation Analysis

Principal components analysis (PCA) was performed to identify associations among bioactive components in chickpea flour (micronutrient concentrations and phytochemical profiles) and observed biological responses. In the preventive conditions, principal components PC1 and PC2 explained 61% of the total variance (Supplementary Figure S5), whereas in the ameliorative conditions, they explained 59.5% (Supplementary Figure S7). These results indicate that the extensive array of variables evaluated (including chickpea micronutrients, phytochemical profile, and biological responses) can be successfully reduced to just two main dimensions (PC1 and PC2) without losing critical information. Likewise, the variable contribution matrix (Supplementary Table S5**)** and Spearman’s correlation analysis (Supplementary Figure S6) under preventive conditions indicate that PC1 is mainly driven by lipid accumulation, with positive contributions from phenolic compounds (malonylated formononetin glycoside (MFG), biochanin A, and formononetin). Likewise, these phenolic compounds were inversely related to interleukin-6 (IL-6) production and glycerol release. Triglyceride accumulation and glutathione peroxidase (GPx) activity showed positive loadings on PC2 and positive contributions from Se and Zn concentrations. The results indicate that under preventive conditions, chickpea phenolic compounds (MFG, biochanin A, and formononetin) effectively counteract inflammation (IL-6) and lipolysis (glycerol release), while micronutrients (Se and Zn) enhance antioxidant defense by increasing GPx activity and could enhance immunomodulatory response.

Unlike in the preventive condition, in the ameliorative model, phenolics MFG, biochanin A, and formononetin shift to share positive loadings on PC2 alongside IL-6 production, glycerol release, and advanced glycation end products (AGE) formation, and show negative associations with lipid accumulation. In contrast, on PC1, lipid accumulation and triglyceride accumulation show positive associations with the phenolic compounds, especially biochanin A and MFG, and an inverse correlation with glycerol release (Supplementary Table S6 and Supplementary Figure S8). Under these conditions, cellular metabolic dynamics are changed by modulating triglyceride metabolism and lipolysis, thereby reducing oxidative stress in steatotic hepatic cells and, hypothetically, maintaining energetic homeostasis and ensuring their survival.

The results indicate that bioactive components of chickpea flour (micronutrients, peptides, and phenolic compounds) do not act through a single pathway; rather, they exhibit complementary and coordinated biological actions. Although individual dose–response effects warrant future investigation, the current work establishes the ingredient’s efficacy.

## 4. Conclusions

Metabolic dysfunction-associated fatty liver disease (MAFLD) disturbances derived from lipid hepatic accumulation are inextricably linked to a pro-inflammatory state and a significant redox imbalance, which are the primary drivers of cellular damage. Zn- and/or Se-biofortified chickpea flours showed significant biological activity that could mitigate the metabolic alterations derived from MAFLD. As a preventive strategy, in HepG2 models, germinated chickpea flour (GC) and biofortified chickpea flour (with Na_2_SeO_3_ or ZnSeO_3_) reduced lipid accumulation and promoted glycerol release, suggesting increased lipolysis. Although ZnSO_4_-biofortified chickpea flour did not decrease lipid accumulation, it appeared to alter cellular energy homeostasis, favoring alternative lipid metabolism pathways, as did a combination of ZnSO_4_ + Na_2_SeO_3_. Moreover, all samples decreased IL-6 release, compared with the positive control (C+). Samples with Se increased glutathione peroxidase (GPx) activity compared with the positive control, as Se is a key component of selenoproteins, including this enzyme. Na_2_SeO_3_ and ZnSeO_3_ increased the activity of GPx beyond that of the negative control (C−). Likewise, the evaluation of ameliorative effects showed significant improvements in multiple markers for certain BCDs. The germinated chickpea flour (GC) and that biofortified with ZnSO_4_ + Na_2_SeO_3_ decreased triglyceride accumulation, possibly by different pathways; while the GC could reduce lipids by increasing lipolysis, biofortified chickpea (with ZnSO_4_+ Na_2_SeO_3_) might do so through potentially favoring an alternative lipid metabolism, such as diminished lipogenesis, and inducing a more metabolically active cell phenotype. Similarly, although ZnSO_4_ BCDs showed no change in triglyceride levels, they showed a significant reduction in lipid levels. Similar to preventive efforts, all samples inhibited IL-6 release relative to C+. Conversely, higher GPx activity was observed in cells treated with Zn, potentially due to enhanced mitochondrial activity and protein synthesis. An improved polyphenol profile and Zn/Se availability via protein binding could activate alternative metabolic pathways, favor compensatory homeostatic mechanisms, and ensure survival. Biofortification, therefore, becomes a sustainable and accessible nutritional alternative for addressing MAFLD, as Se and Zn, alongside the bioactive compounds derived from them, act in a complementary manner to alleviate oxidative stress, inflammation, and impaired lipid metabolism.

It is important to note that these results were obtained using human hepatocellular carcinoma cells (HepG2); they may not fully reflect the response in in vivo steatotic models, so future studies in complex organisms are needed to confirm their effects on these metabolic alterations.

## Figures and Tables

**Table 1:** Methodology for UPLC-PDA-QDa isoflavone detection.

Time	Flow (mL/min)	%A	%B
0.0	0.40	100	0
2.0	0.40	100	0
8.5	0.40	93	7
9.0	0.40	90	10
18.0	0.40	65	35
23.0	0.40	50	50
25.0	0.40	30	70
26.0	0.40	25	75
29.0	0.40	0	100
32.0	0.40	100	0

**Table 2:** Isoflavone content in biofortified chickpea digests (BCD) determined by UPLC-PDA-QDa at 260 nm.

Isoflavone	Concentration (µg/g of BCD)				
	Germinated Control	Na_2_SeO_3_	ZnSO_4_	ZnSeO_3_	ZnSO_4_ + Na_2_SeO_3_
MFG	77.37 ± 5.9 ^b^	82.79 ± 9.3 ^b^	118.37 ± 7.8 ^a^	87.62 ± 7.2 ^b^	80.00 ± 5.4 ^b^
Formononetin	118.05 ± 2.8 ^d^	202.42 ± 5.5 ^a^	211.76 ± 7.2 ^a^	153.59 ± 4.4 ^b^	136.99 ± 4.8 ^c^
Biochanin A	98.40 ± 3.3 ^d^	153.64 ± 5.0 ^b^	211.97 ± 8.9 ^a^	128.88 ± 4.8 ^c^	126.65 ± 4.8 ^c^
**Total**	**293.8 ± 12.0 ^d^**	**438.9 ± 19.7 ^b^**	**542.1 ± 23.8 ^a^**	**370.1 ± 16.3 ^c^**	**343.6 ± 14.9 ^c^**

Data expressed as mean ± SD (n = 3). ^a–d^ Different letters indicate statistically significant differences (*p* < 0.05) amongst treatments in a Tukey’s HSD post hoc test. Isoflavone identification can be found in Supplementary Table S4, which includes the λ max (nm), molecular formula, [M+H]+, and the exact mass of each compound. MFG: Malonylated formononetin glycoside.

## Data Availability

The original contributions presented in this study are included in the article/Supplementary Materials. Further inquiries can be directed to the corresponding author.

## Supplementary Materials

The following supporting information can be downloaded at: https://www.mdpi.com/article/10.3390/foods15132330/s1. Table S1: Salt concentration for chickpea biofortification through germination at industrial scale; Table S2: Composition of simulated salivary fluid (SSF), simulated gastric fluid (SGF), and simulated intestinal fluid (SIF) as final concentration. Summarized from [37]; Table S3: Alpha-amino nitrogen content per sample of biofortified chickpea and biofortified chickpea digests; Table S4: Retention time, λ max (UV max), molecular formula, molecular ion and exact mass for biofortified chickpea flour digests; Table S5: Participation of each variable analyzed in the Principal Component Analysis on each component in a cell model under preventive conditions. Table S6: Participation of each variable analyzed in the Principal Component Analysis on each component in a cell model under ameliorative conditions. Figure S1: Experimental conditions and timing for preventive and ameliorative testing of biofortified chickpea digests (BCDs) in oleic acid-induced HepG2 cells. Figure S2: Preliminary study of cell viability in oleic acid-induced HepG2 cells after exposure to biofortified chickpea digests (BCDs), as (A) prevention, and (B) amelioration. Figure S3: Preliminary study of lipid accumulation via Oil Red O staining in oleic acid-induced HepG2 cells after exposure to biofortified chickpea digests (BCDs), as (A) prevention, and (B) amelioration. Figure S4: Concentrations of zinc (Zn) and selenium (Se) A. Quantifications of Zn and Se in chickpea flour B. Bioaccessibility of Zn and Se in biofortified chickpea digests (BCD) after simulated gastrointestinal digestion. Figure S5: Participation of each variable analyzed in the Principal Component Analysis on each component in a cell model under preventive conditions. Figure S6: Spearman’s correlation heatmap shows associations between phenolic compounds and metabolic biomarkers of metabolic dysfunction and fatty liver disease (MAFLD) in a cell model under preventive conditions. Figure S7: Participation of each variable analyzed in the Principal Component Analysis on each component in a cell model under ameliorative conditions. Figure S8: Spearman’s correlation heatmap shows associations between phenolic compounds and metabolic biomarkers of metabolic dysfunction and fatty liver disease (MAFLD) in a cell model under ameliorative conditions.

## Abbreviations

The following abbreviations are used in this manuscript:

AGE	Advanced glycation end-product
BCD	Biofortified chickpea digest
BSA	Bovine serum albumin
ELISA	Enzyme-linked immunosorbent assay
FTIR	Fourier transformed infrared spectroscopy
FBS	Fetal bovine serum
GPx	Glutathione peroxidase
HbA1c	Glycated hemoglobin
HOMA-IR	Homeostasis model of assessment—estimated insulin resistance
IL-6	Interleukin 6
MAFLD	Metabolic dysfunction-associated fatty liver disease
MASH	Metabolic dysfunction-associated steatohepatitis
MASLD	Metabolic dysfunction-associated steatotic liver disease
MDA	Malondialdehyde
NAFLD	Non-alcoholic fatty liver disease
OA	Oleic acid
PPARγ	Peroxisome proliferator-activated receptor gamma
SDS-PAGE	Sodium dodecyl sulfide polyacrylamide gel electrophoresis
SOD	Superoxide dismutase
SREBP-1c	Sterol Regulatory Element-Binding Protein-1c
