# Supplementary material for: Preventive and Ameliorative Effects of Se- and Zn-Biofortified Chickpeas on MAFLD-Related Metabolic Disturbances
Source: Foods. 2026 Jul 1;15(13):2330. doi: 10.3390/foods15132330 (PMC13361530; doi:10.3390/foods15132330)
Supplement: Supplementary file 1 [file foods-15-02330-s001.zip › foods-4362379-supplementary.pdf]

Preventive and ameliorative effects of Se and Zn biofortified chickpeas on MAFLD-related metabolic disturbances

Emilio López-Millán[1,2], Jorge A. Uribe-Echeverría[1,2], Julián de la Rosa-Millán[1], Marilena Antunes-Ricardo[1,2*]

[1]Tecnologico de Monterrey, School of Engineering and Sciences, Av. Eugenio Garza Sada 2501 Sur, 64849 Monterrey, NL, Mexico

[2]Tecnologico de Monterrey, Institute for Obesity Research, Av. Eugenio Garza Sada 2501 Sur, 64849 Monterrey, NL, Mexico.

*Corresponding author: marilena.antunes@tec.mx (M.A-R)

Supplementary Material

Supplementary Table S1. Salt concentration for chickpea biofortification through germination at an industrial scale

Treatment	Seed/Water ratio	Mineral concentration
Germinated Control		-
Na_2SeO_3		24 mg/L H_2O
$ZnSO_4$	1:3 (w/v) - 166g/L H_2O	24 mg/L H_2O
$ZnSeO_3$		24 mg/L H_2O
$ZnSO_4$ + Na_2SeO_3		12 + 12 mg/L H_2O

Supplementary Table S2. Composition of simulated salivary fluid (SSF) simulated gastric fluid (SGF) and simulated intestinal fluid (SIF) as final concentration. Summarized from Brodkorb et al. (2019)

Component	SSF	SGF	SIF
KCl (mM)	15.1	6.9	6.8
KH_2PO_4 (mM)	3.7	0.9	0.8
$NaHCO_3$ (mM)	13.6	25	85
NaCl (mM)	-	47.2	38.4
$MgCl_2 \cdot 6H_2O$ (mM)	0.15	0.12	0.33
$(NH_4)_2CO_3$ (mM)	0.06	0.5	-
HCl (mM)	-	1.3	8.4
$CaCl_2(H_2O)_2$ (mM)	1.5	0.25	0.6
Salivary amylase (U/mL)	75	-	-
Pepsin (U/mL)	-	2,000	-
Gastric lipase (U/mL)	-	60	-
Bile salts (mM)	-	-	10
Pancreatin (U/mL)	-	-	100

Supplementary Table S3. Alpha amino nitrogen content per sample of biofortified chickpea and biofortified chickpea digests.

Sample type	Treatment	AAN (mg/g sample)
Raw	Germinated Control	4.34 ± 0.04[b]
	Na_2SeO_3	4.75 ± 0.13[a]
	$ZnSO_4$	4.02 ± 0.04[c]
	$ZnSeO_3$	3.32 ± 0.08[d]
	$ZnSO_4$ + Na_2SeO_3	4.63 ± 0.12[a]
Hydrolyzed	Germinated Control	6.30 ± 0.15[A]
	Na_2SeO_3	5.34 ± 0.03[C]
	$ZnSO_4$	5.71 ± 0.02[B]
	$ZnSeO_3$	5.51 ± 0.05[BC]
	$ZnSO_4$ + Na_2SeO_3	5.54 ± 0.26[BC]

AAN = alpha amino nitrogen. Data expressed as mean ± SD (n = 3). [a-d / A-D] Different letters indicate statistically significant differences ($p < 0.05$) amongst treatments using a Tukey test.

Supplementary Table S4. Retention time, λ max (UV max), molecular ion, and exact mass of biofortified chickpea flour digests.

Retention time (min)	Isoflavone	λ max (nm)	Molecular formula	$[M+H]^+$ m/z	Exact mass (Da)
7.7	Malonylated formononetin glycoside (MFG)	248.7	$C_{25}H_{24}O_{12}$	517	516.126
18.1	Formononetin	248.7	$C_{16}H_{12}O_4$	269	268.074
21.6	Biochanin A	259.7	$C_{16}H_{12}O_5$	285	284.068

Supplementary Table S5. Participation of each variable analyzed in the Principal Component Analysis on each component in a cell model under preventive conditions.

Variable	PC1	PC2	PC3	PC4	PC5	PC6	PC7
MFG (µg/g)	0.865	0.261	-0.124	-0.252	-0.196	-0.271	-0.042
Formononetin (µg/g)	0.750	0.395	0.500	0.030	0.001	0.084	0.154
Biochanin A (µg/g)	0.925	0.339	0.120	0.013	-0.060	0.019	0.069
Selenium (µg/g)	-0.374	0.479	0.221	0.649	0.359	-0.164	0.065
Zinc (µg/g)	-0.151	0.522	-0.187	0.533	-0.587	0.194	-0.065
AGE (%)	0.099	-0.584	0.728	0.299	0.021	0.135	-0.005
Lipids accumulation (%)	0.603	0.151	-0.553	0.023	0.478	0.277	-0.075
Triglycerides accumulation (%)	-0.284	0.880	-0.063	-0.328	0.032	0.103	0.105
Glycerol release (%)	-0.675	0.026	0.424	-0.581	-0.066	0.141	0.023
IL-6 production (%)	-0.909	0.201	-0.353	0.049	0.026	-0.061	0.095
GPx activity (%)	-0.212	0.759	0.541	-0.099	0.150	-0.047	-0.230

Selenium and zinc concentrations expressed as µg micronutrient per gram of chickpea flour; IL-6: Interleukin-6, GPx: glutathione peroxidase, AGE: advanced glycation end products, MFG: malonylated formononetin glycoside. MFG, formononetin, and biochanin A are expressed as µg of biochanin A equivalents per gram of chickpea flour. Selenium (Se) and zinc (Zn) are expressed as µg of micronutrient per gram of chickpea flour.

Supplementary Table S6. Participation of each variable analyzed in the Principal Component Analysis on each component in a cell model under ameliorative conditions.

Variable	PC1	PC2	PC3	PC4	PC5	PC6	PC7
MFG (µg/g)	0.746	0.585	-0.073	-0.133	-0.096	-0.268	0.075
Formononetin (µg/g)	0.624	0.469	0.527	0.335	-0.033	0.021	-0.056
Biochanin A (µg/g)	0.729	0.632	0.156	0.193	-0.040	0.029	-0.057
Selenium (µg/g)	-0.059	-0.429	0.042	0.789	0.422	-0.105	-0.023
Zinc (µg/g)	0.195	-0.279	-0.395	0.651	-0.549	0.060	-0.012
AGE (%)	-0.594	0.450	0.577	0.237	-0.045	0.136	0.190
Lipids Accumulation (%)	0.700	-0.697	0.086	0.078	0.011	-0.073	0.150
Triglycerides accumulation (%)	0.669	-0.274	0.641	-0.163	0.084	0.127	-0.058
Glycerol release (%)	-0.883	0.412	-0.126	0.114	-0.041	-0.099	-0.045
IL-6 production (%)	0.227	0.664	-0.572	0.260	0.295	0.100	0.042
GPx activity (%)	0.559	-0.017	-0.779	-0.169	0.107	0.149	0.034

Selenium and zinc concentrations expressed as µg micronutrient per gram of chickpea flour; IL-6: Interleukin-6, GPx: glutathione peroxidase, AGE: advanced glycation end products, MFG: malonylated formononetin glycoside. MFG, formononetin, and biochanin A are expressed as µg of biochanin A equivalents per gram of chickpea flour. Selenium (Se) and zinc (Zn) are expressed as µg of micronutrient per gram of chickpea flour.

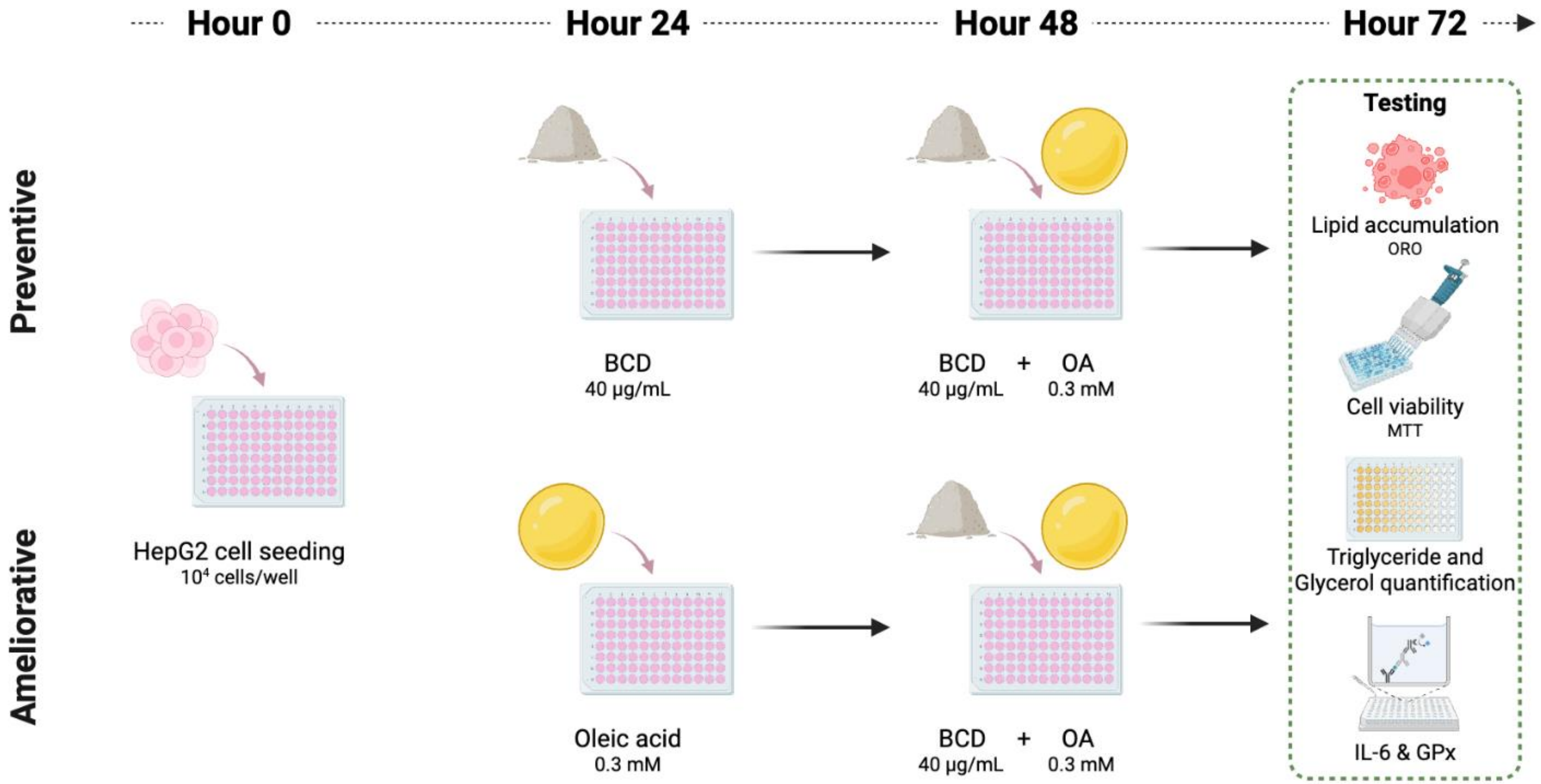

Supplementary Figure S1. Experimental conditions and timing for preventive and ameliorative testing of biofortified chickpea digests (BCDs) in oleic acid-induced HepG2 cells. OA: oleic acid, ORO: Oil Red O, MTS: 3-(4,5-dimethylthiazol-2-yl)-5-(3-carboxymethoxyphenyl)-2-(4-sulfophenyl)-2H-tetrazolium, IL-6: Interleukin-6, GPx: glutathione peroxidase activity.

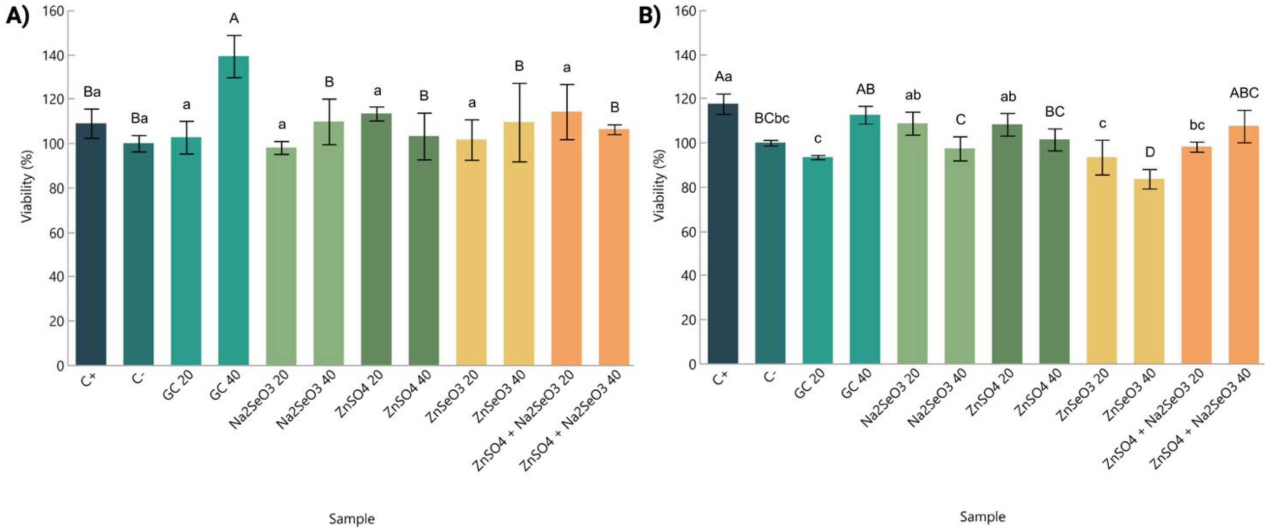

Supplementary Figure S2. Preliminary study of cell viability in oleic acid-induced HepG2 cells after exposure to biofortified chickpea digests (BCDs) as **A)** Prevention, and **B)** Amelioration (n = 3). C+: positive control treated with OA but without BCD, C-: negative control without OA or BCD. [A-C / a - c] Different letters indicate statistically significant differences ($p < 0.05$) amongst treatments in a Tukey's HSD post-hoc test.

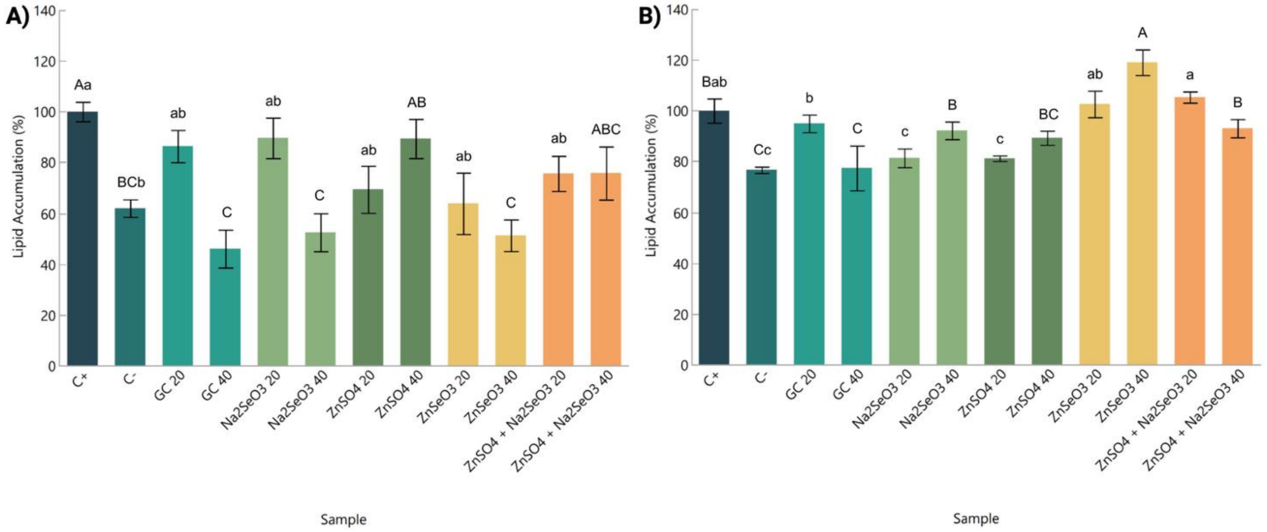

Supplementary Figure S3. Preliminary study of lipid accumulation via Oil Red O staining in oleic acid-induced HepG2 cells after exposure to biofortified chickpea digests (BCDs) as **A)** Prevention, and **B)** Amelioration (n = 3). C+: positive control treated with OA but without BCD, C-: negative control without OA or BCD. [A-C / a - c] Different letters indicate statistically significant differences ($p < 0.05$) amongst treatments in a Tukey's HSD post-hoc test.

A.

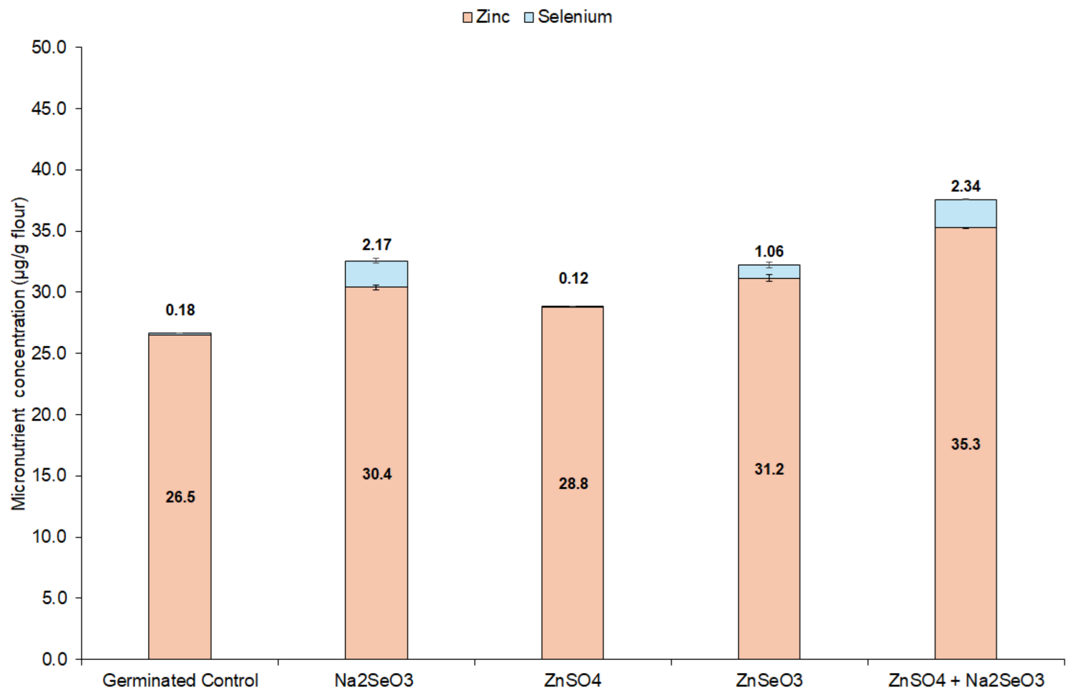

B.

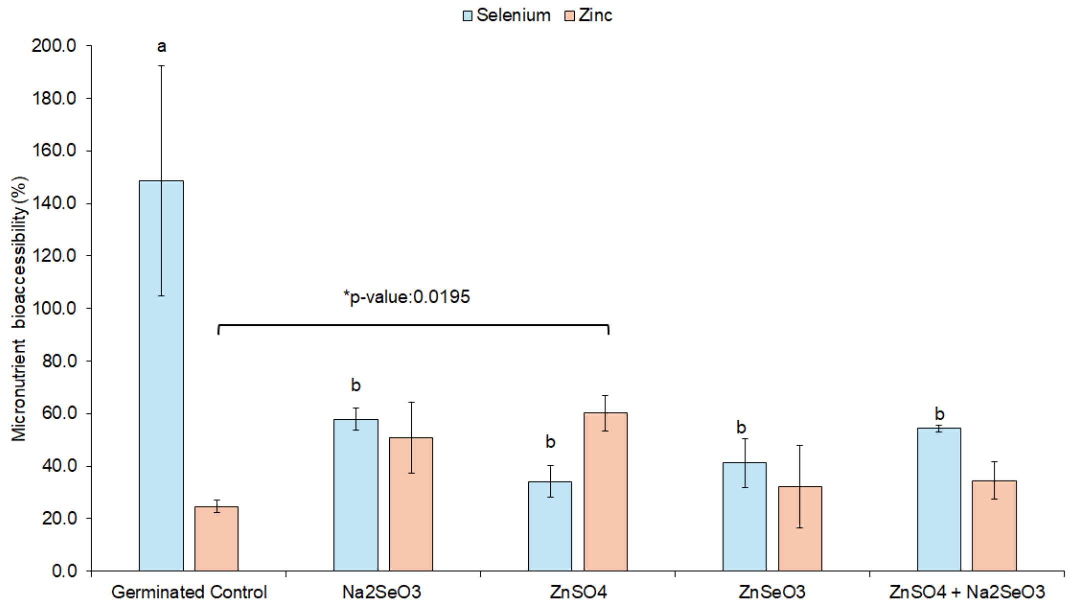

Supplementary Figure S4. Concentration of zinc (Zn) and selenium (Se) **A.** Quantification of Zn and Se in chickpea flour **B.** Bioaccessibility of Zn and Se in biofortified chickpea digests (BCD) after simulated gastrointestinal digestion. [ab] Different letters indicate statistically significant differences ($p < 0.05$) amongst treatments in a Tukey's HSD post-hoc test. *Statistically significant differences ($p < 0.05$) between samples in a two-sample t-test.

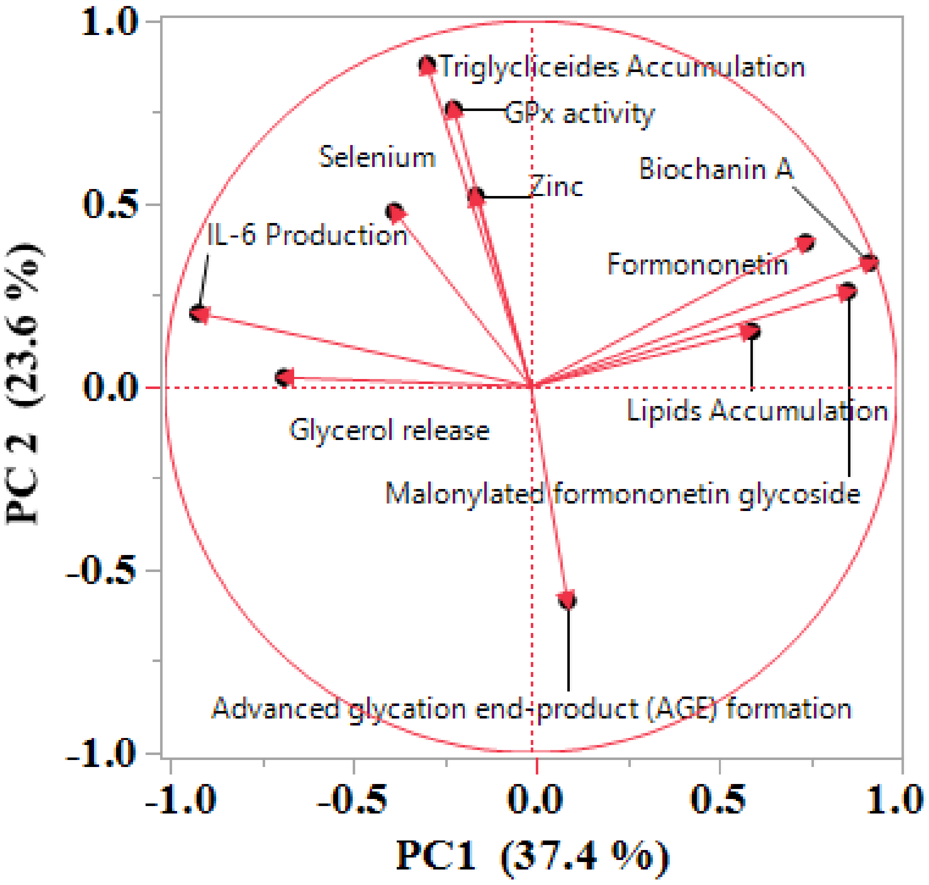

Supplementary Figure S5. Participation of each variable analyzed in the Principal Component Analysis on each component in a cell model under preventive conditions. IL-6: Interleukin-6, GPx: glutathione peroxidase, AGE: advanced glycation end products, MFG: malonylated formononetin glycoside.

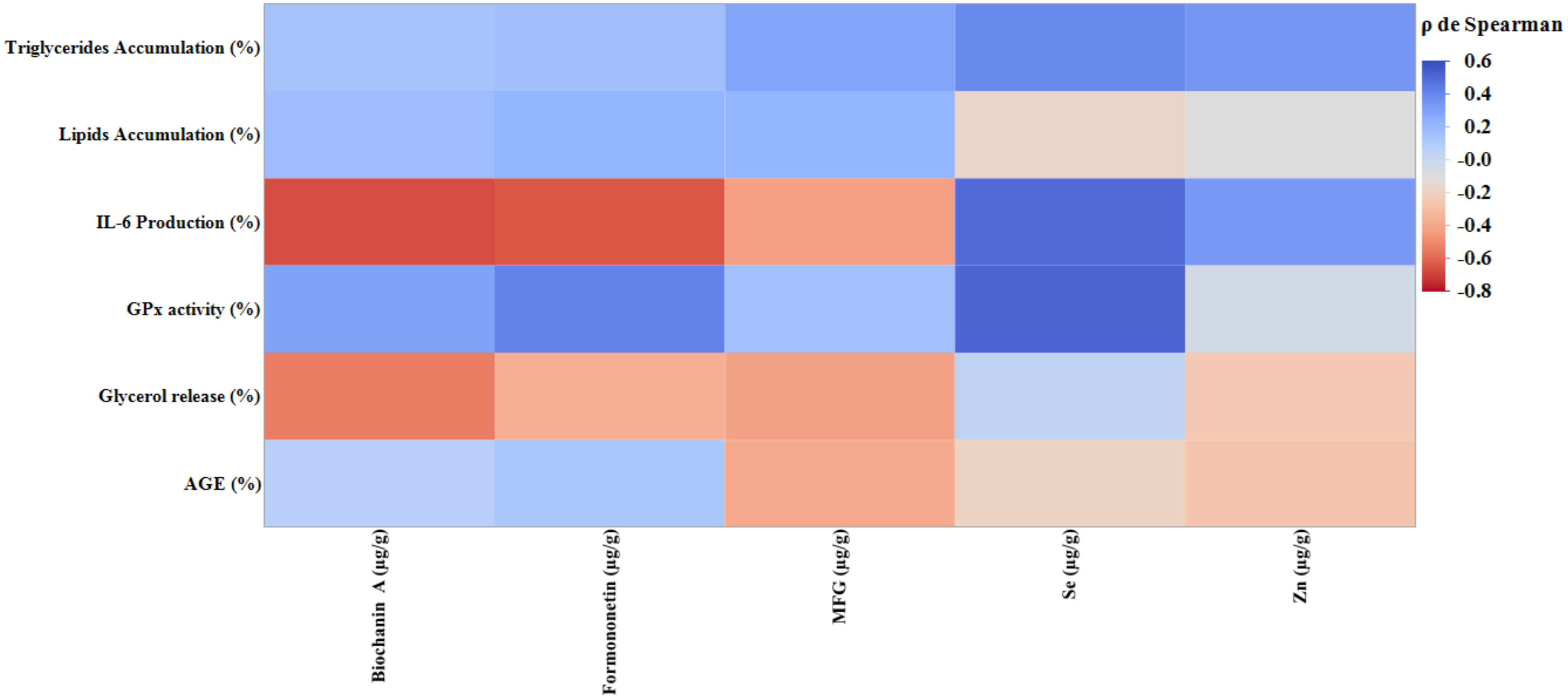

Supplementary Figure S6. Spearman's correlation heatmap shows associations between phenolic compounds and metabolic biomarkers of metabolic dysfunction and fatty liver disease (MAFLD) in a cell model under preventive conditions. IL-6: Interleukin-6, GPx: glutathione peroxidase, AGE: advanced glycation end products, MFG: malonylated formononetin glycoside. Selenium (Se) and zinc (Zn) are expressed as µg of micronutrient per gram of chickpea flour.

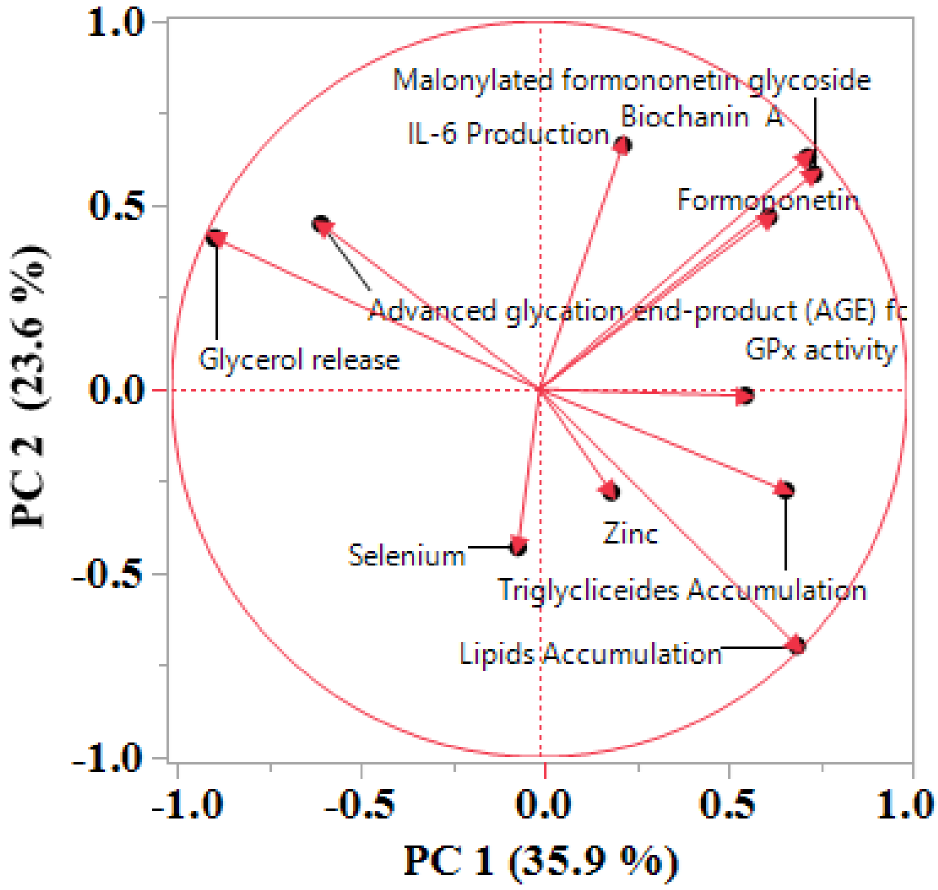

Supplementary Figure S7. Participation of each variable analyzed in the Principal Component Analysis on each component in a cell model under ameliorative conditions. IL-6: Interleukin-6, GPx: glutathione peroxidase, AGE: advanced glycation end products, MFG: malonylated formononetin glycoside.

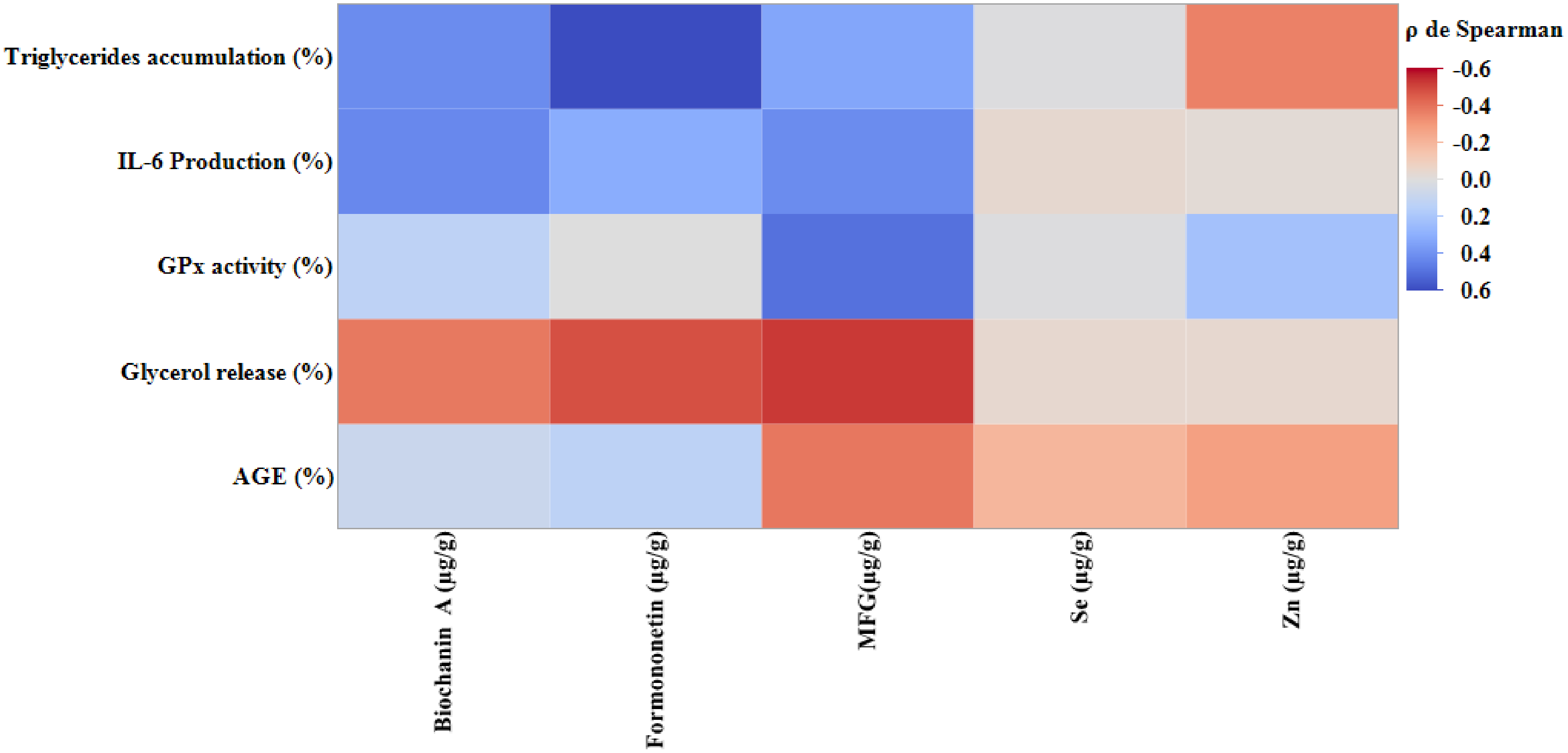

Supplementary Figure S8. Spearman's correlation heatmap shows associations between phenolic compounds and metabolic biomarkers of metabolic dysfunction and fatty liver disease (MAFLD) in a cell model under ameliorative conditions. IL-6: Interleukin-6, GPx: glutathione peroxidase, AGE: advanced glycation end products, MFG: malonylated formononetin glycoside. Selenium (Se) and zinc (Zn) are expressed as µg of micronutrient per gram of chickpea flour.
